# Maternal diabetes-mediated RORA suppression contributes to gastrointestinal symptoms in autism-like mouse offspring

**DOI:** 10.1186/s12868-022-00693-0

**Published:** 2022-02-14

**Authors:** Li Xiao, Min Wang, Wanhua Zhang, Yuan Song, Jiaying Zeng, Huilin Li, Hong Yu, Ling Li, Pingming Gao, Paul Yao

**Affiliations:** 1 Department of Pediatrics, Affiliated Foshan Maternity & Child Healthcare Hospital, The Second School of Clinical Medicine of Southern Medical University, Foshan, 528000 People’s Republic of China; 2 Hainan Women and Children’s Medical Center, Haikou, 570206 People’s Republic of China

**Keywords:** Autism, Gastrointestinal symptoms, Inflammation, Maternal diabetes, Oxidative stress, RORA

## Abstract

**Background:**

Retinoic acid-related orphan receptor alpha (RORA) has been reported to be suppressed in autistic patients and is associated with autism spectrum disorders (ASD), although the potential role and mechanism of RORA on gastrointestinal (GI) symptoms in ASD patients is still not reported. In this study, we aim to investigate the contribution of RORA to GI symptoms through a maternal diabetes-mediated autism-like mouse model.

**Results:**

Male offspring of diabetic dams were treated with either superoxide dismutase (SOD) mimetic MnTBAP or RORA agonist SR1078, or were crossbred with intestine epithelial cells (IEC)-specific RORA knockout (RORA^−/−^) mouse. Gene expression, oxidative stress and inflammation were measured in brain tissues, peripheral blood mononuclear cells (PBMC) and IEC, and GI symptoms were evaluated. Our results showed that SOD mimetic MnTBAP completely, while RORA agonist SR1078 partly, reversed maternal diabetes-mediated oxidative stress and inflammation in the brain, PBMC and IEC, as well as GI symptoms, including intestine permeability and altered gut microbiota compositions. IEC-specific RORA deficiency either mimicked or worsened maternal diabetes-mediated GI symptoms as well as oxidative stress and inflammation in IEC, while there was little effect on maternal diabetes-mediated autism-like behaviors.

**Conclusions:**

We conclude that RORA suppression contributes to maternal diabetes-mediated GI symptoms in autism-like mouse offspring, this study provides a potential therapeutical target for maternal diabetes-mediated GI symptoms in offspring through RORA activation.

**Supplementary Information:**

The online version contains supplementary material available at 10.1186/s12868-022-00693-0.

## Background

Autism spectrum disorders (ASD) are characterized as a group of neurodevelopment disorders with impaired social interaction and communication as well as restricted behaviors that may be triggered by multistage prenatal risk factor exposure [1–3]. Over the past few decades, the prevalence of ASD has increased to around 1:59, with a male/female ratio of 4/1 [2, 3]. A variety of risk factors, including epigenetics and environmental exposures, have been reported to be associated with ASD [4, 5]. We have previously found that prenatal exposure to either maternal diabetes [6–8] or hormones (e.g. progestin and androgen) [9–12] contributes to ASD development through oxidative stress, epigenetic changes and gene suppression [13, 14], while detailed mechanism remains largely unknown.

Accumulating evidence shows that gastrointestinal (GI) symptoms are concurrent and potentially associated with ASD [15]. Gut microbiota may participate in ASD pathogenesis [16], and clinical study shows that microbiota transfer therapy may significantly improve GI and ASD symptoms in autistic patients [17]. Gut microbiota may contribute to brain dysfunction and play a role in ASD development through the gut-brain axis, while the related mechanisms and cause-effect relationship between ASD and GI problems are still unclear [15, 17–20].

Retinoic acid-related orphan receptor alpha (RORA) is involved in many pathophysiological processes [21, 22] and has been shown to be associated with ASD development, with suppressed expression of RORA being identified in autistic patients [23]. Aromatase, encoded by gene cytochrome P450, family 19 (CYP19A1), is the rate-limiting enzyme that is responsible for the conversion of androgen into estradiol (E2) and has been reported to be suppressed in ASD patients and is regulated by RORA either directly or indirectly [24–28]. It has been also reported that RORA regulates the expression of superoxide dismutase 2 (SOD2) through retinoic acid-related orphan receptor elements (RORE) located on the SOD2 promoter [29]. Furthermore, we have early found that maternal diabetes-mediated autism-like mouse offspring has reduced RORA expression in some brain regions as well as in intestinal epithelial cells (IEC). We then hypothesize that RORA may play a role in GI symptoms in maternal diabetes-mediated autism-like mouse offspring through pathways involving the suppression of RORA and its target genes CYP19A1 and SOD2 in the gut system [30, 31].

We have previously found that maternal diabetes-mediated mouse offspring show autism-like behaviors with several core features of ASD, which including decreased ultrasonic vocalizations as well as suppressed social recognition and interaction [8, 32]. In this study, we aim to investigate the potential role of RORA on GI symptoms in maternal diabetes-mediated autism-like mouse models. Our results showed that maternal diabetes-mediated offspring showed significantly suppressed expression of RORA and its target genes CYP19A1 and SOD2 in the brain, peripheral blood mononuclear cells (PBMC) and IEC. The offspring also showed GI symptoms, including oxidative stress, pro-inflammatory cytokine release, increased intestinal permeability and altered gut microbiota composition. Treatment of SOD mimetic MnTBAP [8] completely, while RORA agonist SR1078 [33] partly, reversed this effect. Further experiments using the RORA knockout (RORA^−/−^) mouse model showed that intestine-specific RORA deficiency mimicked or worsened maternal diabetes-mediated oxidative stress and inflammation in IEC as well as GI symptoms. We conclude that RORA suppression contributes to maternal diabetes-mediated GI symptoms in mouse offspring.

## Results

### RORA agonist and SOD mimetic reverse maternal diabetes-mediated RORA suppression and oxidative stress in the brain

**Fig. 1:**
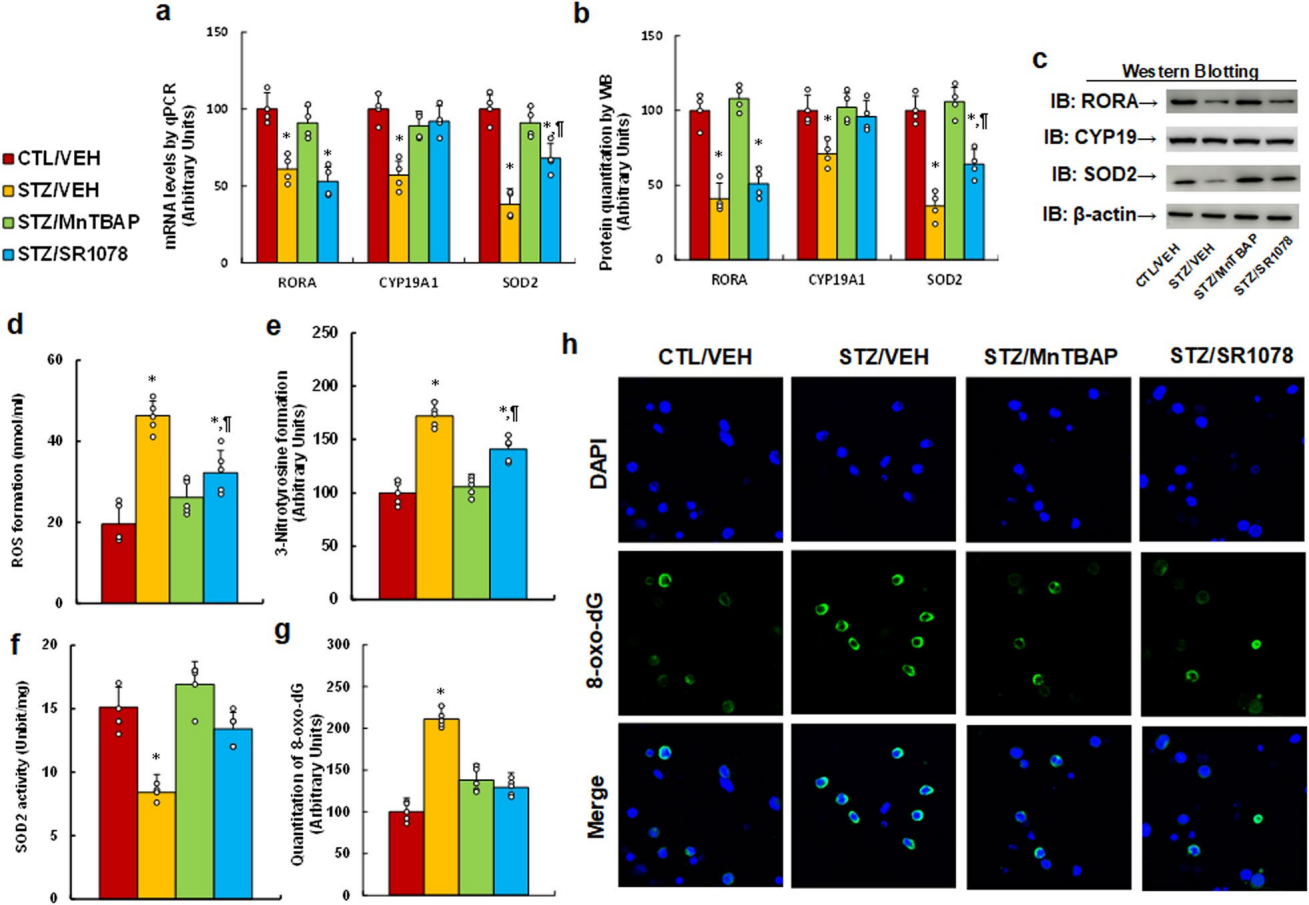
RORA agonist and SOD mimetic reverse maternal diabetes-mediated RORA suppression and oxidative stress in the amygdala. Dams were divided into the following treatment groups: control (CTL/VEH), diabetes (STZ/VEH), diabetes plus SOD mimetic MnTBAP (STZ/MnTBAP), and diabetes plus RORA agonist SR1078 (STZ/SR1078) during pregnancy, and the amygdala neurons or tissues were isolated from male offspring for biomedical analysis. **a** mRNA levels by qPCR, n = 4. **b** Quantitation of protein levels, n = 5. **c** Representative pictures of western blotting for **b**, and full-length blots are presented in Additional file 1: Fig. S1a. **d** ROS formation, n = 5. **e** 3-Nitrotyrosine formation, n = 5. **f** SOD2 activity, n = 5. **g**, **h** The amygdala neurons were isolated on embryonic day (E18) from the above treatment for immunostaining. **g** Quantitation of 8-oxox-dG staining, n = 5. **h** Representative pictures for 8-oxo-dG staining (green) and DAPI staining for nuclei (blue). The one-way ANOVA analysis was performed to determine statistical significance of different groups. *P < 0.05, vs. CTL/VEH group; ^¶^*P* < 0.05, vs. STZ/VEH group. Data were expressed as mean ± SD

Dams were treated by control (CTL/VEH), diabetes (STZ/VEH), diabetes plus SOD mimetic MnTBAP (STZ/MnTBAP), or diabetes plus RORA agonist SR1078 (STZ/SR1078) during pregnancy. The amygdala neurons and brain tissues, including the amygdala, hypothalamus and hippocampus, were isolated from subsequent male offspring for biomedical analysis. We first evaluated the gene expression of RORA and its target genes from amygdala tissues. The results showed that maternal diabetes (STZ/VEH) treatment significantly deceased the mRNA levels of RORA, CYP19A1 and SOD2 (*P* < 0.0001), respectively, compared to the CTL/VEH group; SOD mimetic MnTBAP treatment (STZ/MnTBAP) completely reversed this effect; while RORA agonist (STZ/SR1078) completely reversed the expression of CYP19A1, partly reversed on SOD2 (*P* < 0.01), but showed no effect on RORA (see Fig. 1a). We also measured the protein levels by western blotting, and an expression pattern similar with that of mRNA levels was observed (see Fig. 1b, c, Additional file 1: Fig. S1a). We then evaluated oxidative stress, and the results showed that maternal diabetes (STZ/VEH) significantly increased ROS formation (see Fig. 1d) and 3-nitrotyrosin formation (see Fig. 1e) (*P* < 0.0001), respectively, compared to CTL/VEH group. STZ/MnTBAP treatment completely, while RORA agonist (STZ/SR1078) partly, reversed this effect. We also evaluated SOD2 activity, and the results showed that maternal diabetes (STZ/VEH) significantly decreased SOD2 activity (*P* < 0.0001) compared to the CTL/VEH group; treatments of either STZ/MnTBAP or STZ/SR1078 completely reversed this effect (see Fig. 1f). Finally, we evaluated 8-oxo-dG formation from amygdala neurons. The results showed that maternal diabetes (STZ/VEH) significantly increased 8-oxo-dG formation (*P* < 0.0001) compared to the CTL/VEH group; treatments of either STZ/MnTBAP or STZ/SR1078 had no significant effect (see Fig. 1g, h). In addition, we evaluated gene expression in the hypothalamus and hippocampus, and the results showed that maternal diabetes (STZ/VEH) significantly decreased expression of RORA and CYP19A1 in both the hypothalamus and hippocampus compared to the CTL/VEH group. STZ/MnTBAP treatment completely reversed this effect on both genes, while STZ/MnTBAP treatment completely reversed this effect on CYP19A1 expression but had no effect on RORA. Additionally, none of the treatments had any effect on SOD2 expression in tissues of the hypothalamus and hippocampus (see Additional file 1: Fig. S2). We conclude that RORA agonist and SOD mimetic reverse maternal diabetes-mediated RORA suppression and oxidative stress in the brain.

### RORA agonist and SOD mimetic ameliorate maternal diabetes-mediated autism-like behaviors in male offspring

**Fig. 2:**
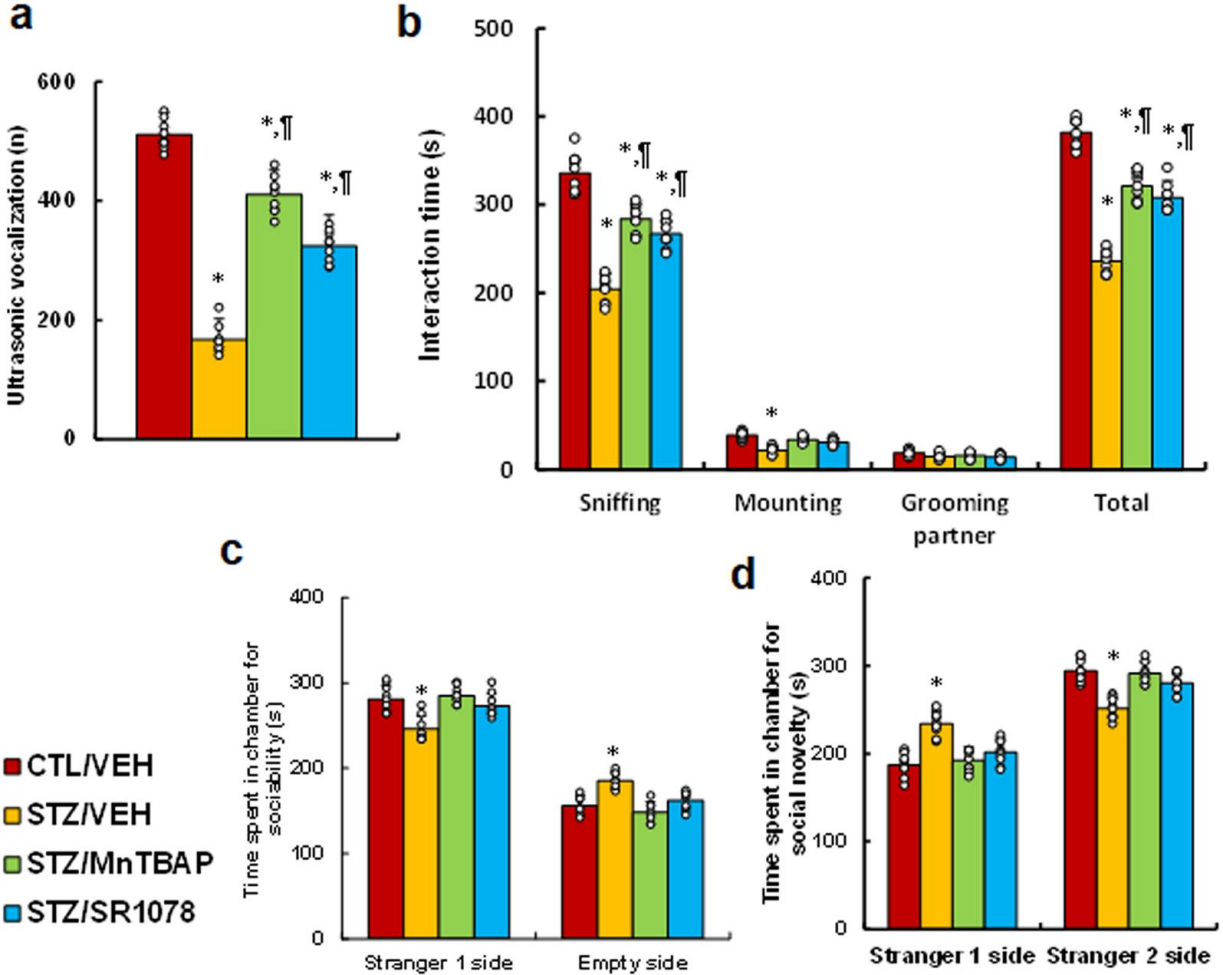
RORA agonist and SOD mimetic ameliorate maternal diabetes-mediated autism-like behaviors in male offspring. Dams were divided into the following treatment groups: control (CTL/VEH), diabetes (STZ/VEH), diabetes plus SOD mimetic MnTBAP (STZ/MnTBAP), or diabetes plus RORA agonist SR1078 (STZ/SR1078) during pregnancy, and the subsequent male offspring were used for evaluation of autism-like behaviors. **a** Ultrasonic vocalization, n = 9. **b** Social interaction (SI) test, with total interaction time and amount of time spent following, mounting, grooming, and sniffing any body parts of the other mouse were calculated, n = 9. **c**, **d** Three-chambered social tests, n = 9. **c** Time spent in chamber for sociability. **d** Time spent in chamber for social novelty. The one-way ANOVA analysis was performed to determine statistical significance of different groups. *P < 0.05, vs. CTL/VEH group; ^¶^*P* < 0.05, vs. STZ/VEH group. Data were expressed as mean ± SD

We evaluated the effects of RORA agonist and SOD mimetic MnTBAP on maternal diabetes-mediated autism-like behaviors. Our result showed that offspring from the maternal diabetes (STZ/VEH) treatment group displayed significantly decreased ultrasonic vocalizations (*P* < 0.0001) compared to CTL/VEH treatment; both SOD mimetic (STZ/MnTBAP) or RORA agonist (STZ/SR1078) partly reversed this effect (*P* < 0.01, see Fig. 2a). Additionally, mice in the maternal diabetes (STZ/VEH) group spent significantly less time sniffing (*P* < 0.0001), mounting (*P* < 0.01), and interacting in total (*P* < 0.0001) during the social interaction tests compared to the CTL/VEH group; both STZ/MnTBAP and STZ/SR1078 treatment partly reversed the effect on sniffing (*P* < 0.01) and total interaction time (*P* < 0.01) while completely reversing the effect on mounting interaction time. None of the treatments appeared to have a significant effect on the amount of time spent grooming partners (see Fig. 2b). Furthermore, mice from the maternal diabetic (STZ/VEH) treatment group spent significantly less time in the stranger 1 side (*P* < 0.01) and more time in the empty side (*P* < 0.01) during the sociability test (see Fig. 2c); while spent significant more time in the stranger 1 side (*P* < 0.01) and less time in the stranger 2 side (*P* < 0.01) in the social novelty test compared to the CTL/VEH treatment group (see Fig. 2d); both STZ/MnTBAP and STZ/SR1078 treatment completely reversed the effect. We conclude that RORA agonist and SOD mimetic ameliorate maternal diabetes-mediated autism-like behaviors in male offspring.

### RORA agonist and SOD mimetic reverse maternal diabetes-mediated pre-inflammatory cytokine release in PBMC

**Fig. 3:**
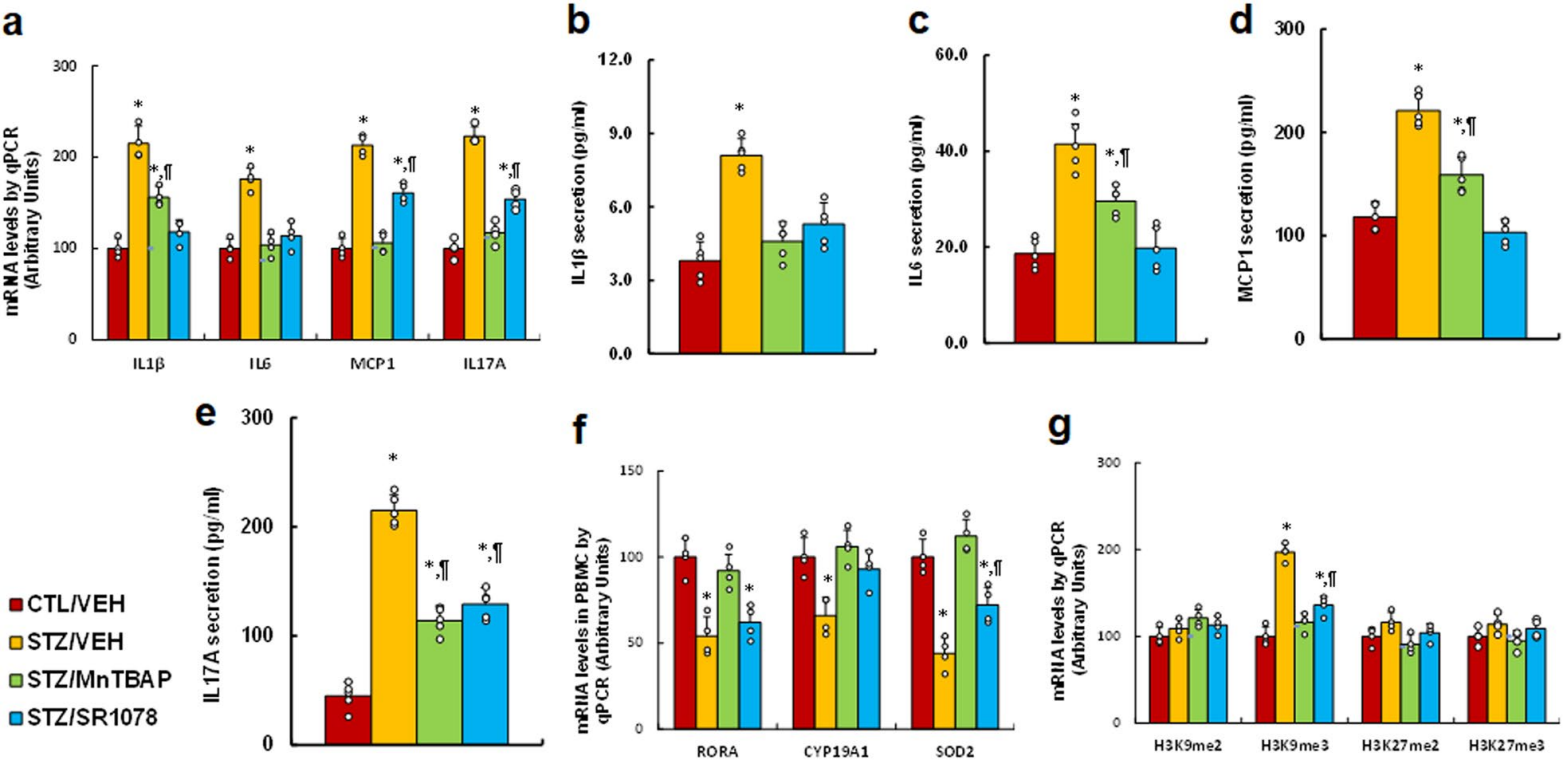
RORA agonist and SOD mimetic reverse maternal diabetes-mediated pre-inflammatory cytokine release in PBMC. Dams were divided into the following treatment groups: control (CTL/VEH), diabetes (STZ/VEH), diabetes plus SOD mimetic MnTBAP (STZ/MnTBAP), or diabetes plus RORA agonist SR1078 (STZ/SR1078) during pregnancy, and the PBMC were isolated from male offspring for biomedical analysis. **a** mRNA levels of pre-inflammatory cytokines, n = 4. **b** IL-1β secretion, n = 9. **c** IL-6 secretion, n = 9. **d** MCP1 secretion, n = 9. **e** IL17A secretion, n = 9. **f** mRNA levels of RORA and target genes, n = 4. **g** ChIP analysis on the RORA promoter, n = 4. The one-way ANOVA analysis was performed to determine statistical significance of different groups. *P < 0.05, vs. CTL/VEH group; ^¶^*P* < 0.05, vs. STZ/VEH group. Data were expressed as mean ± SD

We evaluated the potential effect of RORA agonist on pre-inflammatory cytokine release in PBMC cells. Our results showed that maternal diabetes (STZ/VEH) treatment significantly increased mRNA levels of IL1β, IL6, MCP1 and IL17A (*P* < 0.0001), respectively, compared to the CTL/VEH treatment; SOD mimetic (STZ/MnTBAP) treatment completely reversed the effects on the expression of IL6, MCP1 and IL17A, while partly reversed the effects on IL1β expression (*P* < 0.001). RORA agonist (STZ/SR1078) completely reversed the effects on the expression of IL1β and IL6 and partly reversed the effects on MCP1 (*P* < 0.001) and IL17A (*P* < 0.01) expression (see Fig. 3a). We also measured the cytokine secretion in cultured PBMC, which included IL1β (see Fig. 3b), IL6 (see Fig. 3c), MCP1 (see Fig. 3d) and IL17A (see Fig. 3e). The results showed that maternal diabetes (STZ/VEH) treatment significantly increased pro-inflammatory cytokine secretion compared to the CTL/VEH treatment (*P* < 0.0001), and treatments of STZ/MnTBAP and STZ/SR1078 either completely or partly reversed the effects of maternal diabetes. We further evaluated the gene expression of RORA and its target genes in PBMC, and the results showed that maternal diabetes (STZ/VEH) treatment significantly decreased mRNA levels of RORA and its target genes CYP19A1 and SOD2 (*P* < 0.0001) compared to CTL/VEH treatment, and STZ/MnTBAP treatment completely, while STZ/SR1078 treatment partly, reversed this effect (see Fig. 3f). In addition, we evaluated the potential effect of maternal diabetes on epigenetic changes on the RORA promoter in PBMC cells, and the results showed that the different treatments had no effect on DNA methylation (see Additional file 1: Fig. S3), Histone 4 (H4) methylation (see Additional file 1: Fig. S4a), or histone acetylation (see Additional file 1: Fig. S4b). Finally, we measured histone 3 methylation on the RORA promoter, and the results showed that the maternal diabetes (STZ/VEH) treatment group displayed significantly increased H3K9me3 modification (*P* < 0.0001) compared to the CTL/VEH treatment. STZ/MnTBAP treatment completely, while STZ/SR1078 treatment partly reversed this effect (*P* < 0.01). The treatments had no effect on H3K9me2, H3K27me2 or H3K27me3 modifications (see Fig. 3g). We conclude that RORA agonist and SOD mimetic reverse maternal diabetes-mediated pre-inflammatory cytokine release in PBMC cells.

### RORA agonist and SOD mimetic reverse maternal diabetes-mediated RORA suppression and oxidative stress in IEC

**Fig. 4:**
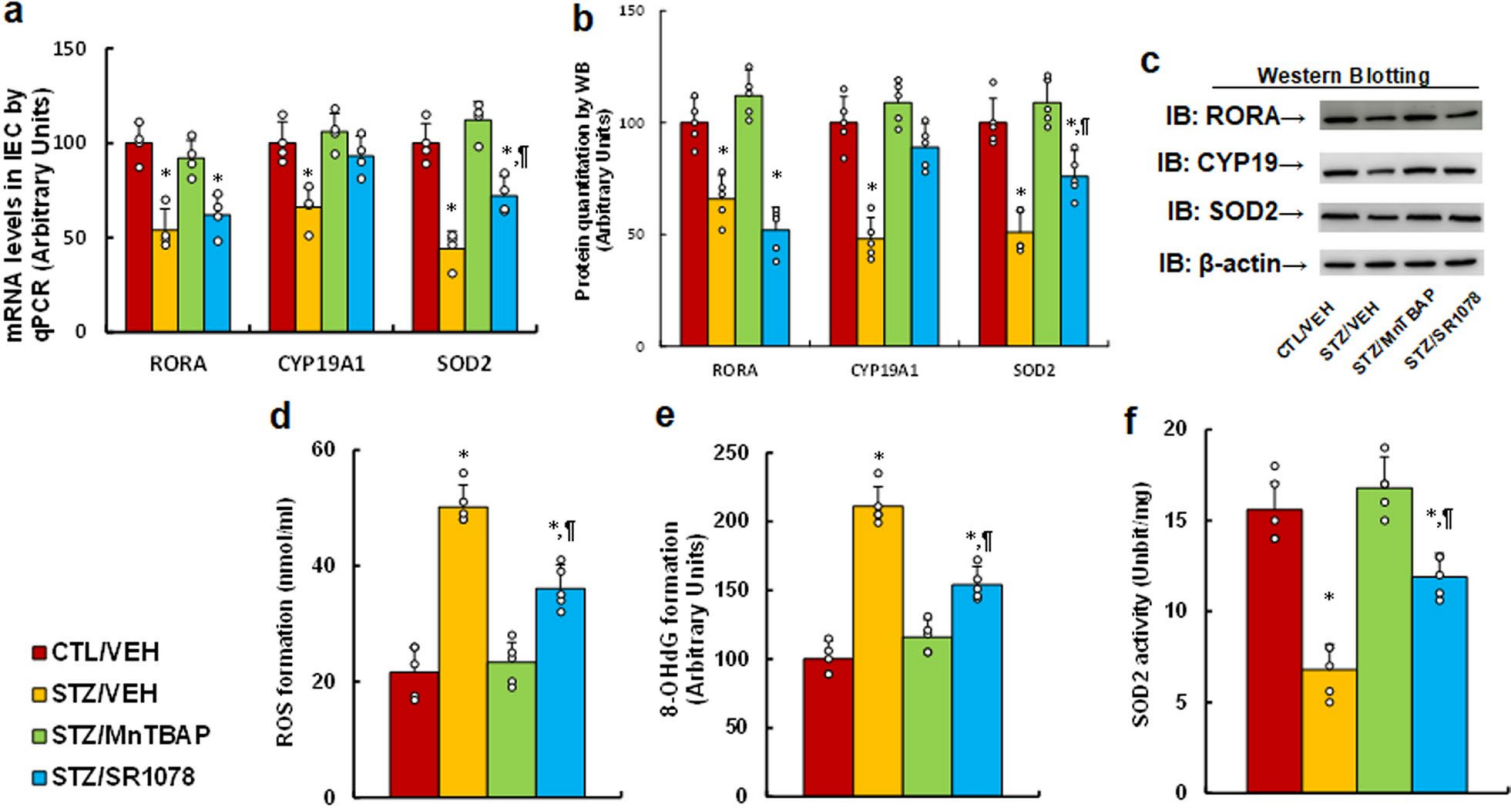
RORA agonist and SOD mimetic reverse maternal diabetes-mediated RORA suppression and oxidative stress in IEC. Dams were divided into the following treatment groups: control (CTL/VEH), diabetes (STZ/VEH), diabetes plus SOD mimetic MnTBAP (STZ/MnTBAP), or diabetes plus RORA agonist SR1078 (STZ/SR1078) during pregnancy, and the IEC cells were isolated from male offspring for biomedical analysis. **a** mRNA levels by qPCR, n = 4. **b** Quantitation of protein levels, n = 5. **c** Representative pictures of western blotting for **b**, and full-length blots are presented in Additional file 1: Fig. S1b. **d** ROS formation, n = 5. **e** 8-OHdG formation, n = 5. **f** SOD2 activity, n = 5. The one-way ANOVA analysis was performed to determine statistical significance of different groups. *P<0.05, vs. CTL/VEH group; ^¶^*P*<0.05, vs. STZ/VEH group. Data were expressed as mean ± SD

We evaluated the potential effect of maternal diabetes and RORA agonist on gene expression and oxidative stress in IEC cells. The results showed that maternal diabetes (STZ/VEH) significantly decreased the mRNA levels of RORA, CYP19A1 and SOD2 (*P* < 0.0001) compared to the CTL/VEH group; MnTBAP treatment (STZ/MnTBAP) completely, while STZ/SR1078 treatment partly (*P* < 0.01), reversed this effect (see Fig. 4a). We also measured the protein levels by western blotting, and an expression pattern similar with that of mRNA levels was observed (see Fig. 4b, c, Additional file 1: Fig. S1b). We then evaluated oxidative stress, and the results showed that maternal diabetes (STZ/VEH) significantly increased ROS formation (see Fig. 4d) and 3-nitrotyrosin formation (see Fig. 4e), respectively, compared to the CTL/VEH group (*P* < 0.0001); STZ/MnTBAP treatment completely, while RORA agonist (STZ/SR1078) partly, reversed this effect. We finally evaluated the SOD2 activity, and the results showed that offspring in the maternal diabetes (STZ/VEH) group showed decreased SOD2 activity (*P* < 0.0001) compared to the CTL/VEH group; again, STZ/MnTBAP treatment completely, while RORA agonist (STZ/SR1078) partly (*P* < 0.01), reversed this effect (see Fig. 4f). We conclude that RORA agonist and SOD mimetic reverse maternal diabetes-mediated RORA suppression and oxidative stress in IEC.

### RORA agonist and SOD mimetic reverse maternal diabetes-mediated gastrointestinal dysfunction

**Fig. 5:**
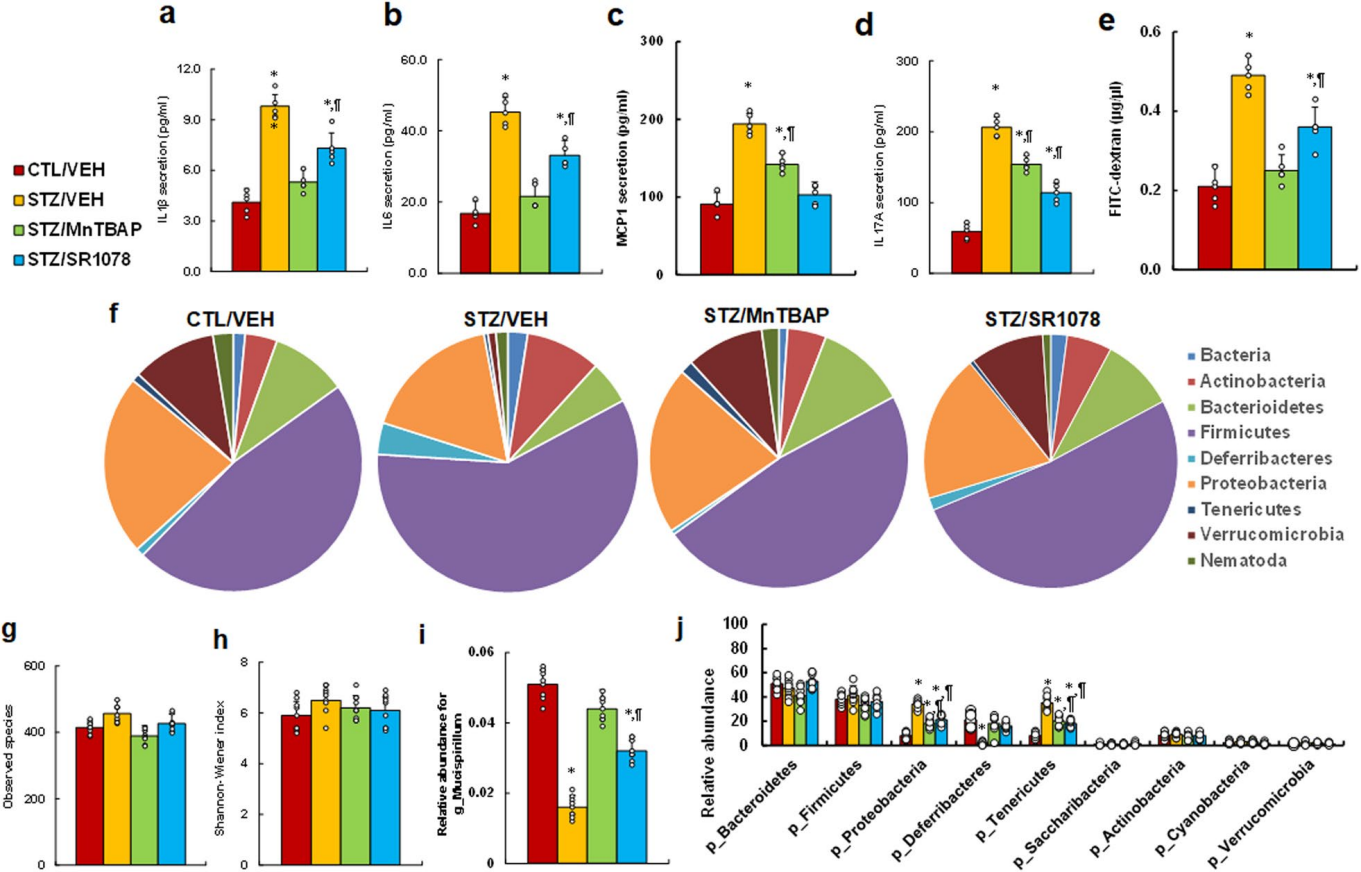
RORA agonist and SOD mimetic reverse maternal diabetes-mediated gastrointestinal dysfunction. Dams were treated by control (CTL/VEH), diabetes (STZ/VEH), diabetes plus SOD mimetic MnTBAP (STZ/MnTBAP), or diabetes plus RORA agonist SR1078 (STZ/SR1078) during pregnancy, and GI functions of male offspring were evaluated. **a**–**d** The IEC cells were isolated to evaluate cytokine secretion, n = 9; **a** IL-1β secretion; **b** IL-6 secretion; **c** MCP1 secretion; **d** IL17A secretion. **e** Intestinal permeability assay by FITC-dextran, n = 5. **f**–**j** Gut microbiota analysis, n = 9; **f** overview of the identified relative frequencies of different phyla found in treated mice; **g** species richness; **h** species diversity; **i** relative abundance of Mucispirillumn at genus level; **j** relative abundance of different bacteria in phylum level. The one-way ANOVA analysis was performed to determine statistical significance of different groups. *P < 0.05, vs. CTL/VEH group; ^¶^*P* < 0.05, vs. STZ/VEH group. Data were expressed as mean ± SD

We evaluated the potential effect of maternal diabetes and RORA agonist on gastrointestinal function. First, we measured cytokine secretion from isolated IEC cells, and the results showed that maternal diabetes (STZ/VEH) significantly increased cytokine secretion of IL1β (see Fig. 5a), IL6 (see Fig. 5b), MCP1 (see Fig. 5c) and IL17A (see Fig. 5d) compared to the CTL/VEH group (*P* < 0.0001); treatments of STZ/MnTBAP and STZ/SR1078 partly reversed this effect. We then evaluated GI function by intestinal permeability assay using FITC-dextran, and the results showed that maternal diabetes (STZ/VEH) significantly increased intestinal permeability (*P* < 0.0001) compared to the CTL/VEH group; STZ/MnTBAP treatment completely, while STZ/SR1078 treatment partly (*P* < 0.01), reversed this effect (see Fig. 5e). We then evaluated the microbial populations of the treated mice using 16S rRNA gene sequencing, and the results showed that maternal diabetes (STZ/VEH) resulted in significant differences in gut microbial composition compared to CTL/VEH group. In general, the phyla Firmicutes and Proteobacteria dominate the microbiome of CTL/VEH mice, while a shift towards Firmicutes and Actinobacteria occurred in STZ/VEH mice; and treatments of both STZ/MnTBAP and STZ/SR1078 partly reversed this effect (see Fig. 5f). Additionally, there was no difference in microbial species richness (see Fig. 5g) and diversity (see Fig. 5h) across the treatment groups. Furthermore, we evaluated the relative abundance of different gut organisms. The genus level of Mucispirillum (g_Mucispirillum) was significantly decreased in the STZ/VEH treatment group (*P* < 0.0001) compared to the CTL/VEH group; STZ/MnTBAP treatment completely, while STZ/SR1078 treatment partly (*P* < 0.01), reversed this effect (see Fig. 5i). In addition, a genus belonging to the phylum Deferribacteres (p_Deferribacteres) was significantly decreased in STZ/VEH treatment compared to the CTL/VEH group, and treatments of STZ/MnTBAP and STZ/SR1078 completely reversed this effect. On the other hand, genus belonging to the phylum Proteobacteria (p_Proteobacteria) (*P* < 0.001) and Tenericutes (p_Tenericutes) (*P* < 0.001) were significantly increased in the STZ/VEH treatment group compared to the CTL/VEH group; treatments of STZ/MnTBAP and STZ/SR1078 partly reversed this effect (see Fig. 5j). In general, our results indicate that RORA agonist and SOD mimetic reverse maternal diabetes-mediated gastrointestinal dysfunction, including cytokine release, increased intestinal permeability and changes in gut microbial composition.

### Intestine-specific RORA deficiency mimics or worsens maternal diabetes-mediated RORA suppression and oxidative stress in IEC

**Fig. 6:**
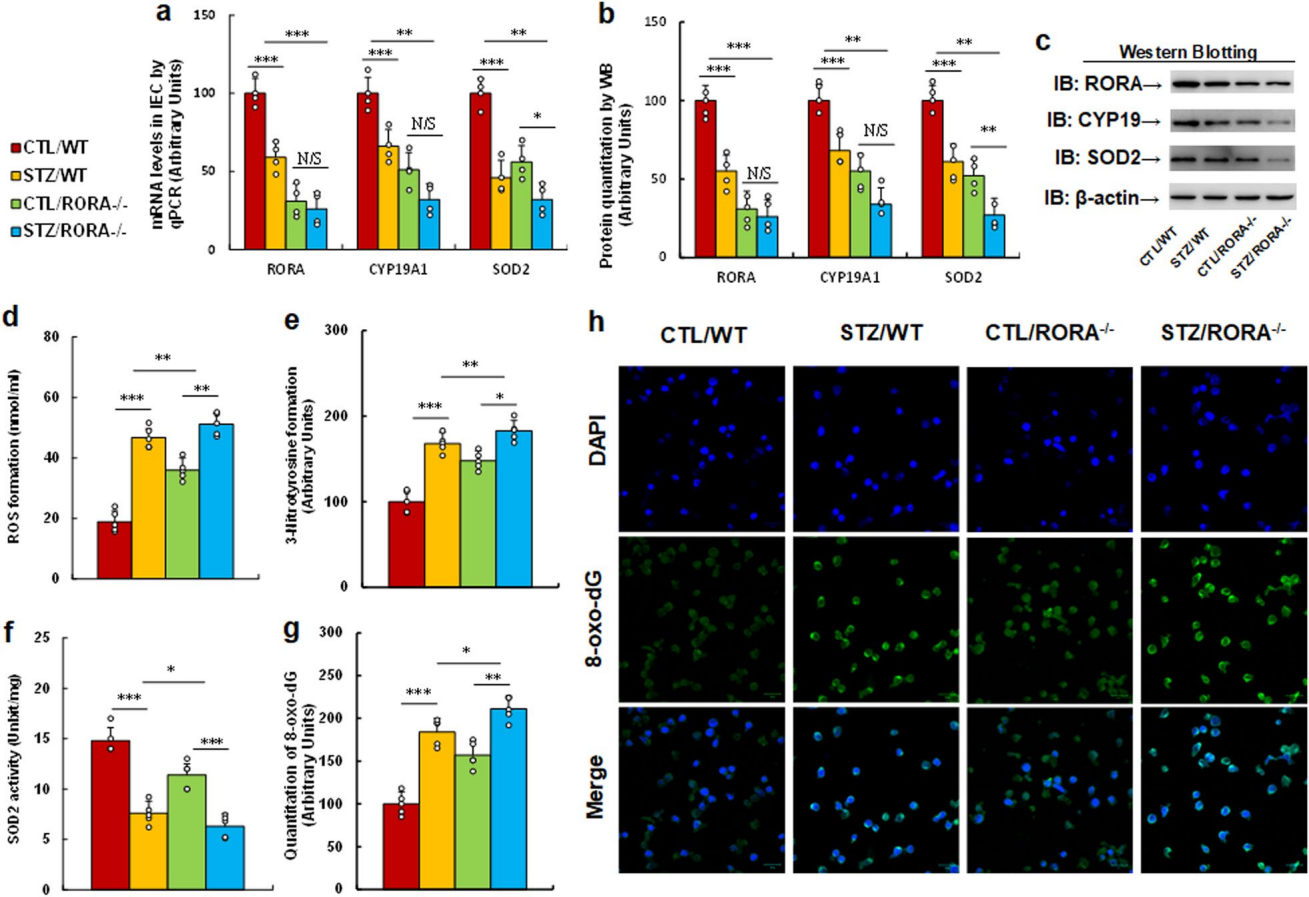
Intestine-specific RORA deficiency mimics or worsens maternal diabetes-mediated RORA suppression and oxidative stress in IEC. Dams in either the control (CTL) or diabetes (STZ) groups were crossbred with either wild type (WT) or intestinal epithelial-specific RORA knockout (RORA^−/−^) mice, and the IEC cells were isolated from subsequent male offspring for biomedical analysis. **a** mRNA levels by qPCR, n = 4. **b** Quantitation of protein levels, n = 5. **c** Representative pictures of western blotting for **b**, and full-length blots are presented in Additional file 1: Fig. S1c. **d** ROS formation, n = 5. **e** 3-Nitrotyrosine formation, n = 5. **f** SOD2 activity, n = 5. **g**, **h** The IEC were isolated for 8-oxo-dG immunostaining; **g** representative pictures for 8-oxo-dG staining (green) and DAPI staining for nuclei (blue); **h** quantitation of 8-oxox-dG staining, n = 5; The two-way ANOVA analysis was performed to determine statistical significance of different groups. ***P < 0.0001; **P < 0.001; *P < 0.01; N/S, no significance. Data were expressed as mean ± SD

Dams in either the control (CTL) or diabetes (STZ) groups were crossbred with either wild type (WT) or intestinal epithelial-specific RORA knockout (RORA^−/−^) mice, and the IEC cells were isolated from subsequent male offspring for biomedical analysis. We first evaluated gene expression from IEC, and the results showed that maternal diabetes (STZ/WT) significantly decreased mRNA levels of RORA, CYP19A1 and SOD2, respectively, compared to the CTL/WT group. RORA deficiency (RORA^−/−^), including treatments of CTL/RORA^−/−^ and STZ/RORA^−/−^, either mimicked or worsened this effect (see Fig. 6a). We also measured the protein levels by western blotting, and an expression pattern similar to that of mRNA levels was observed (see Fig. 6b, c, Additional file 1: Fig. S1c). We then evaluated oxidative stress, and the results showed that maternal diabetes (STZ/WT) significantly increased ROS formation (see Fig. 6d) and 3-nitrotyrosin formation (see Fig. 6e), respectively, compared to the CTL/WT group; RORA deficiency (RORA^−/−^) either mimicked or worsened this effect. We then evaluated SOD2 activity, and the results showed that maternal diabetes (STZ/WT) significantly decreased SOD2 activity compared to the CTL/WT group (see Fig. 6f); we then evaluated 8-oxo-dG formation by immunostaining in IEC, and the results showed that maternal diabetes (STZ/WT) significantly increased 8-oxo-dG formation compared to the CTL/WT group (see Fig. 6g, h); again, RORA deficiency (RORA^−/−^) either mimicked or worsened these effects. We conclude that intestine-specific RORA deficiency mimics or worsens maternal diabetes-mediated RORA suppression and oxidative stress in IEC.

### Intestine-specific RORA deficiency does not affect maternal diabetes-mediated autism-like behaviors

We evaluated the potential effect of intestine-specific RORA deficiency on maternal diabetes-mediated gene expression and autism-like behaviors. Our results showed that maternal diabetes (STZ/WT) treatment significantly decreased the mRNA levels of RORA, CYP19A1 and SOD2 in the amygdala compared to the CTL/WT group, and intestine-specific RORA deficiency (RORA^−/−^) showed no effect on gene expression (see Additional file 1: Fig. S5a). We then evaluated autism-like behaviors in those animals, and the results showed that maternal diabetes (STZ/WT) treatment significantly decreased ultrasonic vocalizations (see Additional file 1: Fig. S5b). Moreover, offspring from the STZ/WT group spent significantly less time interacting with stranger mice during the social interaction tests (see Additional file 1: Fig. S5c) and showed significant less interest in sociability (see Additional file 1: Fig. S5d) and social novelty (see Additional file 1: Fig. S5e) compared to the CTL/WT group. RORA deficiency (RORA^−/−^), including treatments of CTL/RORA^−/−^ and STZ/RORA^−/−^, showed no effect on these behaviors. We conclude that intestine-specific RORA deficiency does not affect maternal diabetes-mediated autism-like behaviors.

### Intestine-specific RORA deficiency mimics or worsens maternal diabetes-mediated gastrointestinal dysfunction

**Fig. 7:**
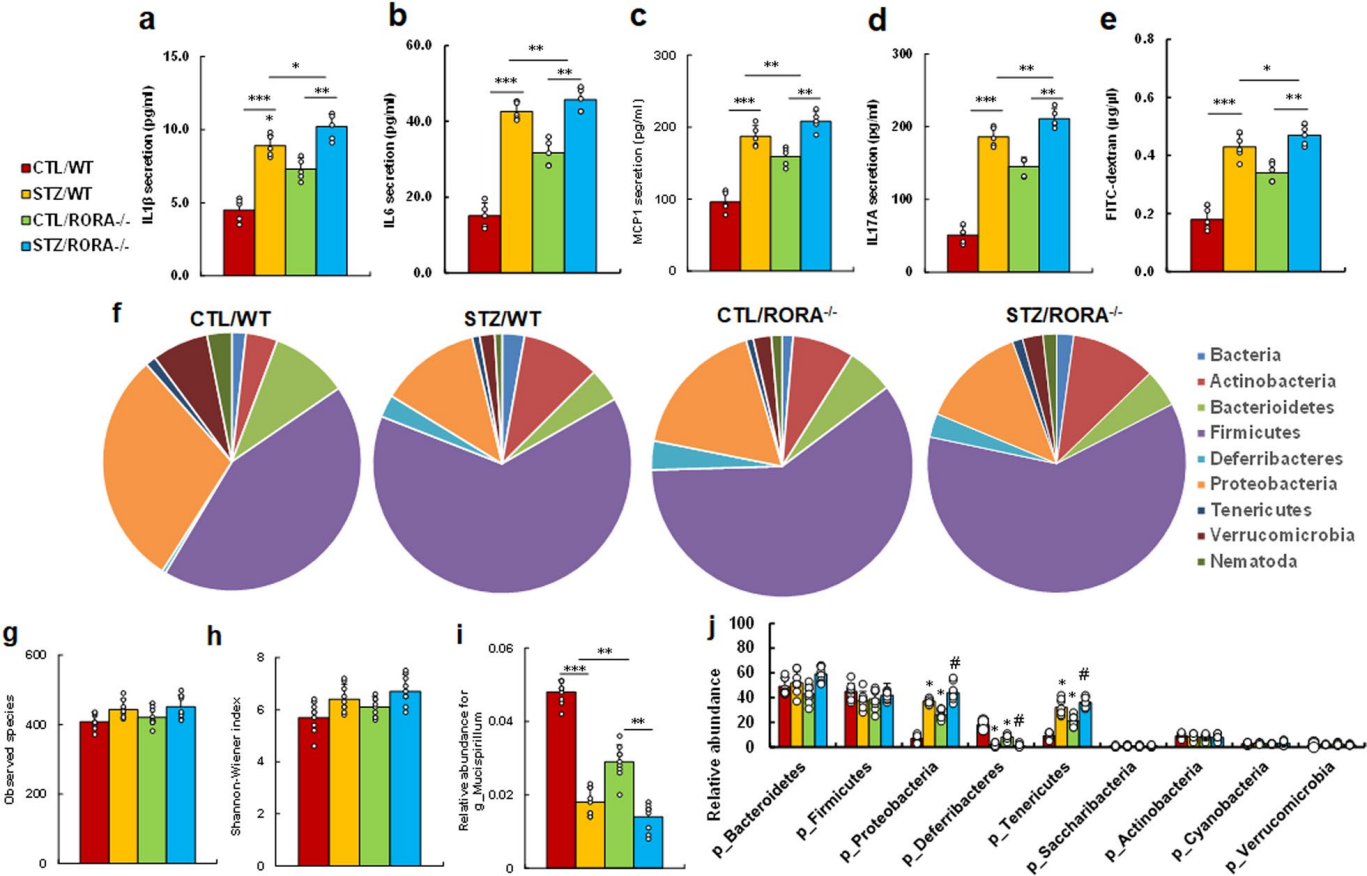
Intestine-specific RORA deficiency mimics or worsens maternal diabetes-mediated gastrointestinal dysfunction. Dams from either the control (CTL) or diabetes (STZ) groups were crossbred with either wild type (WT) or intestinal epithelial-specific RORA knockout (RORA^−/−^), and the gastrointestinal function of male offspring was evaluated. **a**–**d** The IEC cells were isolated to evaluate cytokine secretion, n = 9; **a** IL-1β secretion; **b** IL-6 secretion; **c** MCP1 secretion; **d** IL17A secretion. **e** Intestinal permeability assay by FITC-dextran, n = 5. **f**–**j** Gut microbiota analysis, n = 9; **f** overview of the identified relative frequencies of different phyla found in treated mice; **g** species richness; **h** species diversity; **i** relative abundance of Mucispirillumn at genus level; **j** relative abundance of different bacteria in phylum level. The two-way ANOVA analysis was performed to determine statistical significance of different groups. ***P < 0.0001; **P < 0.001; *P < 0.01; N/S, no significance. Data were expressed as mean ± SD

We evaluated the potential effect of RORA deficiency on maternal diabetes-mediated gastrointestinal dysfunction. We first measured cytokine secretion from isolated IEC cells, and the results showed that maternal diabetes (STZ/WT) significantly increased cytokine secretion of IL1β (see Fig. 7a), IL6 (see Fig. 7b), MCP1 (see Fig. 7c) and IL17A (see Fig. 7d) compared to CTL/WT group; RORA deficiency (RORA^−/−^), including treatments of CTL/RORA^−/−^ and STZ/RORA^−/−^, either mimicked or worsened this effect. We then evaluated GI function by intestinal permeability assay, and the results showed that maternal diabetes (STZ/WT) significantly increased intestinal permeability compared to CTL/WT group; RORA deficiency (RORA^−/−^) either mimicked or worsened this effect (see Fig. 7e). We then evaluated the microbial populations of the treated mice using 16S rRNA gene sequencing, and the results showed that maternal diabetes (STZ/WT) treatment achieved significant differences in gut microbial composition compared to the CTL/WT group; in general, the phyla Firmicutes and Proteobacteria dominate the microbiome of CTL/WT mice, while a shift towards Firmicutes and Actinobacteria occurred in STZ/WT mice; RORA deficiency (RORA^−/−^) mimicked the effect of STZ/WT treatment (see Fig. 7f). Again, there was no difference in the microbial species richness (see Fig. 7g) and diversity (see Fig. 7h) among those treatments. Furthermore, we evaluated the relative abundance of different gut organisms. The genus level of Mucispirillum (g_Mucispirillum) was significantly decreased in the STZ/WT treatment group compared to the CTL/WT group; RORA deficiency (RORA^−/−^) either mimicked or worsened this effect (see Fig. 7i). In addition, there were significantly fewer organisms of a genus belonging to the phylum Deferribacteres (p_Deferribacteres) in STZ/WT treatment compared to the CTL/WT group; RORA deficiency (RORA^−/−^) either mimicked or worsened this effect. On the other hand, the number organisms of a genus belonging to the phylum Proteobacteria (p_Proteobacteria) and Tenericutes (p_Tenericutes) was significantly increased in the STZ/WT treatment group compared to CTL/WT group; and RORA deficiency (RORA^−/−^) either mimicked or worsened this effect (see Fig. 7j). In general, our results indicate that intestine-specific RORA deficiency mimics or worsens maternal diabetes-mediated gastrointestinal dysfunction, including cytokine release, increased intestinal permeability and changes of the gut microbial composition.

### Schematic model for maternal diabetes-mediated GI symptoms through RORA suppression in autism-like offspring

**Fig. 8:**
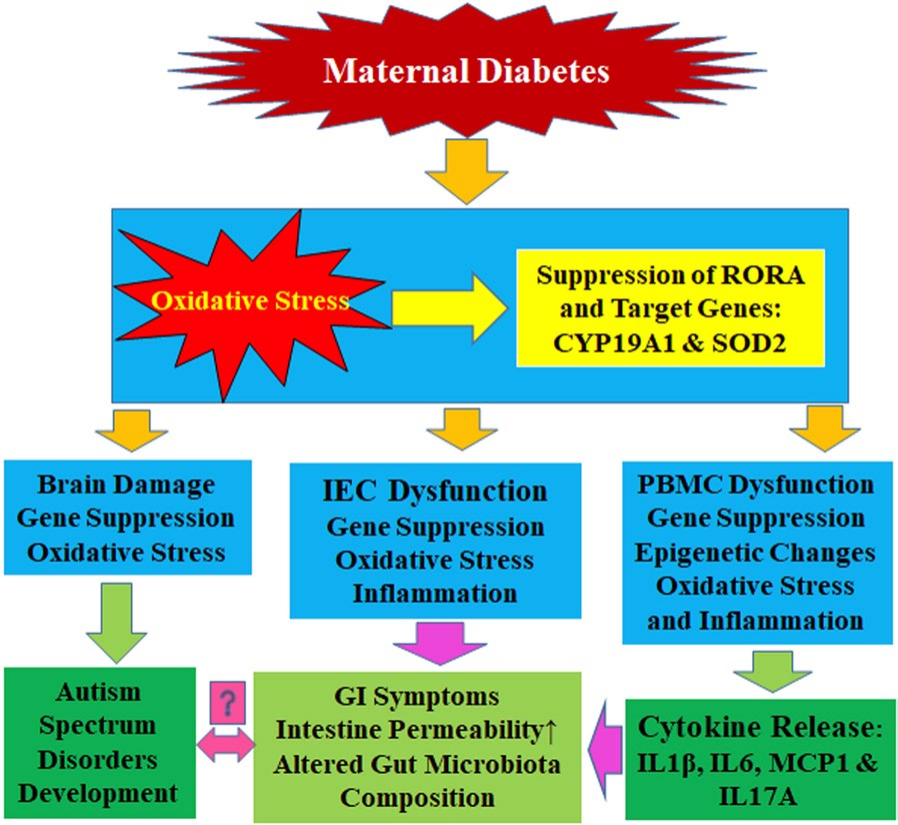
Schematic model for maternal diabetes-mediated GI symptoms through RORA suppression in autism-like offspring. *ASD* autism spectrum disorders, *CYP19A1* cytochrome P450, family 19 (aromatase), *IEC* intestine epithelial cells, *GI* gastrointestinal, *IL1β* interleukin-1β, *MCP1* monocyte chemoattractant protein-1, *PBMC* peripheral blood mononuclear cells, *RORA* retinoic acid-related orphan receptor α, *SOD2* superoxide dismutase 2

Maternal diabetes-mediated oxidative stress [34] triggers suppression of RORA and its target genes (CYP19A1 and SOD2) in the brain, leading to ASD development. Simultaneously, maternal diabetes-induces RORA suppression by epigenetic changes on the RORA promoter in PBMC, resulting in pro-inflammatory cytokine release, which including IL1β, IL6, MCP1 and IL17A. Furthermore, maternal diabetes-mediated RORA suppression in IEC leads to inflammation, increased intestinal permeability and altered gut microbial composition, subsequently resulting in GI symptoms. On the other hand, the cause-effect relationship between ASD and GI symptoms in maternal diabetes-mediated mouse model remains unclear (see Fig. 8).

## Discussion

In this study, we found that maternal diabetes-mediated autism-like mouse offspring have suppressed expression of RORA and its target gene in addition to oxidative stress and inflammation in brain tissues, PBMC and IEC, as well GI symptoms. RORA agonist SR1078 partly reversed this effect, and intestine-specific RORA deficiency mimics or worsens maternal diabetes-mediated GI symptoms.

### RORA suppression in autism-like behaviors

It has previously been reported that RORA expression is reduced in the brains of ASD patients [23], while its potential role in ASD development is still largely unknown. Our results showed that RORA expression was significantly suppressed in brain tissues of maternal diabetes-mediated autism-like mouse models. SOD mimetic MnTBAP completely, while RORA agonist SR1078 partly, reversed maternal diabetes-mediated autism-like behaviors, indicating that maternal diabetes-mediated oxidative stress plays a major role [8]. This effect is at least partly mediated through RORA and its target genes, including CYP19A1 and SOD2, since CYP19A1 converts androgen into estradiol, which directly or indirectly regulates many antioxidant genes [35, 36]. RORA may play an important role in sex bias in ASD development [37] by modulating the expression of CYP19A1 and SOD2 as well as oxidative stress in neurons [8, 28].

### RORA suppression in PBMC and its contribution to GI symptoms

Our results showed that maternal diabetes-mediated autism-like offspring had significant GI symptoms, together with suppressed RORA expression in PBMC [38] and increased secretion of proinflammatory cytokines, including IL1β, IL6 [39], MCP1 and IL17A [40]. It has been reported that altered gut microbiota composition in ASD is associated with systemic immune dysfunction, such as chronic inflammation [38], and RORA regulates the inflammatory signaling pathway by modulation of macrophage development and activation [41, 42]. We suppose that maternal diabetes-mediated RORA suppression in PBMC may contribute to GI symptoms in autism-like offspring by modulation of systemic inflammation in PBMC cells.

### RORA suppression in IEC and its contribution to GI symptoms

We found that maternal diabetes-mediated autism-like offspring had RORA suppression in IEC with significant GI symptoms [43–45], including oxidative stress and inflammation in IEC [46], increased intestine permeability and altered microbiota compositions. SOD mimetic MnTBAP completely, while RORA agonist SR1078 partly, reversed this effect. This indicates that RORA suppression may play an important role in maternal diabetes-mediated GI symptoms, although this does not exclude other potential factors. Furthermore, IEC-specific RORA deficiency mimicked or worsened maternal diabetes-mediated GI symptoms, while surprisingly, it showed little effect on maternal diabetes-mediated autism-like behaviors. It has been widely reported that gut microbiota may modulate animal behaviors through the gut-brain axis and fecal microbiota transplantation (FMT) [47] rescues social behaviors in ASD model [48, 49]. Our results indicate that IEC may not play a major role in gut microbiota-mediated animal behaviors, while it has been reported that gut microbiota may modulate autism-like behaviors through the vagus nerve or enteric nervous system [50, 51], and this may be the potential explanation for our findings.

### Limitations

We have previously showed that the amygdala plays a major role in maternal diabetes-mediated autism-like behavior [6–8]; therefore, gene expression in the amygdala was a key focus in this study, while this does not exclude the potential contribution and effect from other brain area on animal behaviors. Furthermore, only male offspring were studied here in order to avoid potential interference from estrogen/estrogen receptor-mediated effects in female offspring [6–8], and this may potentially affect the conclusions about the role of the estrous cycle in the assessment of animal behaviors and GI symptoms. Additionally, the lack of IEC-specific RORA expression mouse is a potential limitation to investigate the mechanism of RORA and its contribution to GI symptoms.

### Conclusions

Maternal diabetes-mediated autism-like mouse offspring display RORA suppression in the brain, PBMC and IEC with oxidative stress and inflammation. They also show a variety of GI symptoms, including increased intestine permeability and altered gut microbiota composition. Treatment with either SOD mimetic or RORA agonist partly reverses this effect. Furthermore, IEC-specific RORA deficiency either mimics or worsens maternal diabetes-mediated GI symptoms. We conclude that RORA suppression contributes to maternal diabetes-mediated GI symptoms in autism-like mouse offspring, this study provides a potential therapeutical target for maternal diabetes-mediated GI symptoms in offspring through RORA activation.

## Methods

A detailed description can be found in Additional file 1: Data S1, and the related primers used in this study are shown in Additional file 1: Table S1.

### Reagents and materials

The antibodies for β-actin (sc-47778), CYP19 (sc-374176), RORA (sc-518081) and SOD2 (sc-30080) were obtained from Santa Cruz Biotechnology. Fluorescein isothiocyanate-labeled dextran (FITC-dextran, #46944) and RORA agonist SR1078 (#557352) were obtained from Sigma.

### In vivo mouse experiments

The animal protocol conformed to US NIH guidelines (Guide for the Care and Use of Laboratory Animals, No. 85-23, revised 1996), and was reviewed and approved by the Institutional Animal Care and Use Committee from Foshan Maternity and Child Health Care Hospital and Hainan Women and Children’s Medical Center, and this study is in accordance with the ARRIVE guidelines. In this study, 10 dams were assigned for each exposure, and one representative offspring was picked up randomly from each dam for experiments and analysis, and 9 representative offspring were usually picked up from 10 in total in case animal may die by accident during experimental process. Each animal was firstly used for behavior analysis, then was sacrificed for tissue collection and subsequent biological analysis. Each experimental offspring was in separate cage since they came from different dams.

#### Generation of intestine-specific RORA knockout mice

The RORA^fl/fl^ mouse, which has loxP flanking sites targeting exon 3 of the RORA gene, was generated by in vitro fertilization and was obtained for the study as a generous gift from Dr. Haimou Zhang from Hubei University. The Villin-cre (Vil1-cre) transgenic mice (#021504) have the mouse villin 1 promoter directing expression of Cre recombinase to villus and crypt epithelial cells of the small and large intestines, was obtained from Jackson Laboratories. To generate intestine-specific RORA^−/−^ null mouse (Vil1-cre-RORA^fl/fl^), the RORA^fl/fl^ mice were cross-bred with Vil1-cre mice for over 4 generations on the C57BL/6J background. Positive offspring were confirmed by genotyping through PCR using specific primers (see Additional file 1: Table S1) for the presence of both loxP sites within RORA alleles and Cre recombinase [22].

#### Generation of diabetic mice

All the experimental mice were either RORA wild type (WT) or RORA null (RORA^−/−^) mice with a C57BL/6J mixed genetic background. Chronic diabetic female mice were induced by injection of 35 mg/kg streptozotocin (STZ), and the diabetic mice were confirmed with blood glucose > 250 mg/dl.

#### Mouse protocol 1: prenatal treatment of diabetes or chemicals

Verified pregnant dams (n = 9 for each group) were randomly assigned to the following 4 groups: Group 1: CTL group mice receiving only vehicle treatment (CTL/VEH); Group 2: STZ mice receiving only vehicle treatment (STZ/VEH); Group 3: CTL group mice receiving 10 mg/kg/day of MnTBAP (dissolved in DMSO) injection (CTL/MnTBAP); Group 4. STZ mice receiving 10 mg/kg/day of RORA agonist SR1078 (dissolved in DMSO) injection (STZ/SR1078). The injection was conducted on days 1, 4, 7, 10, 13, 16 and 19 of pregnancy, respectively. Neurons from the amygdala were isolated on embryonic day 18 (E18) as described below. The male offspring (1 male offspring from each dam was randomly selected for experiments) were separated from the dams on day 21, fed with normal chow, and underwent behavior tests until 7–8 weeks of age. GI symptoms were evaluated, the whole blood was collected by heart puncture and the peripheral blood mononuclear cells (PBMC) were isolated, and the intestine epithelial cells (IEC) were isolated as described below for further biomedical analysis,

#### Mouse protocol 2: prenatal treatment of diabetes or RORA deficiency

Verified pregnant dams (n = 9 for each group) were randomly assigned to the following 4 groups. Group 1: CTL group mice with RORA WT background (CTL/WT); Group 2: STZ mice with RORA WT background (STZ/WT); Group 3: CTL group mice with intestine-specific RORA knockdown background (CTL/RORA^−/−^); Group 4: STZ mice with intestine-specific RORA knockdown background (STZ/RORA^−/−^). The subsequent offspring were used for analysis as described in “Mouse protocol 1: prenatal treatment of diabetes or chemicals”.

### Animal behavior test

The animal behavior test of offspring was usually carried out at 7–8 weeks of age unless otherwise indicated. Autism-like behavior was evaluated using ultrasonic vocalization (USV), social interaction (SI) tests and a three-chambered social test [52–54], and detailed methods are described in Additional file 1.

### Isolation of mouse intestine epithelial cells (IEC)

The protocol for isolation of IEC cells was based on the previously described method with minor modifications. In brief, the small and large intestines were harvested individually from treated mice and rinsed extensively with RPMI-1640 media (from Lonza) after Peyer’s patches were removed (for small intestine). The rinsed intestines were opened longitudinally and macerated; the tissue pieces were shaken gently in RPMI-1640 containing 2 mM EDTA and 10% fetal calf serum. The tissue preparations were passed through 70-µm mesh filters, and the resulting single-cell suspensions were applied to Percoll (from Sigma) density gradients of 25%, 40%, and 75%. After centrifugation at 2000×*g* for 20 min, the interface between the 25% and 40% layers was collected to obtain IECs. The cells were stained using antibodies for either epithelial cell adhesion molecule (EpCAM, from Biolegend) or CD45 (from Biolegend) and nucleic acid dye (Via-Probe, from BD Biosiences). The Via-Probe^−^/CD45^−^/EpCAM^+^ IEC were sorted using BD FACSMelody^TM^ Cell Sorter (BD Biosciences) for further biomedical analysis [55, 56].

### Intestinal permeability assay

The protocol was followed based on the previously described method with minor modifications. In brief, treated mice were fasted for 4 h before the experiment and then the FITC-dextran (50 mg/mL, Cat# 46944 from Sigma) was gavaged to mice (600 mg/kg). After 4 h, the whole blood was collected by cardiac puncture and placed at room temperature for 1 h before being centrifuged at a speed of 3000 rpm for 10 min. The supernatant was then transferred to a new tube for further centrifugation at a speed of 12,000 rpm for 10 min at 4 °C. The subsequent supernatant (serum) was diluted with equal volume of PBS and 100 µL diluted serum was added to a 96-cell microplate. The concentration of FITC in serum was determined at excitation/emission wavelengths of 485/530nm using a FLx800 microplate fluorescence reader (Bio-Tek). The serial diluted FITC-dextran (0, 0.5, 1, 2, 4, 6, 8, 10 µg/µL) was used as standards. Serum of mice administered with PBS was used as negative controls [17, 57].

### Fecal microbiome analysis

Fecal samples of the experimental mice were collected and stored at − 80 °C before being processed. The microbial DNA was extracted using a QIAamp Fast DNA Stool Mini Kit (from Qiagen) according to the manufacturer’s protocol [58]. The purity and concentration of the extracted DNA were detected using agarose gel electrophoresis. Fecal microbiota was studied by performing V3–V4 16S rDNA amplicon sequencing in order to obtain the operational taxonomic units (OTU) defining the bacterial communities [59]. Sequencing samples from frozen fecal pellets were prepared, sequenced and subsequently processed using the MiSeq Pe300 Sequencing Platform (from Illumina) by Shanghai OE Biotech Inc. The raw data were treated and processed using a QIIME 2^TM^ software package, and the subsequent sequences of OTU were blasted in the Silva database (version 138). The alpha diversity and beta diversity were analyzed using QIIME 2^TM^ software package [17].

### Analysis of cytokines

Mouse cytokines from either the serum or cell supernatant, including IL1β, IL6, IL17a and MCP1 were measured using the Bio-Plex Pro Mouse Cytokine 23-plex Assay kit (#M60009RDPD from BioRad) and Bio-Plex 200 Systems (BioRad) according to the manufacturer’s instructions [60].

### Statistical analysis

The one-way analysis of variance (ANOVA) followed by the Tukey−Kramer test was used to determine statistical significance of different groups in “Mouse protocol 1: prenatal treatment of diabetes or chemicals”, and the two-way ANOVA followed by the Bonferroni post hoc test was used to determine the differences of two factors (e.g. RORA deficiency and maternal diabetes) in “Mouse protocol 2: prenatal treatment of diabetes or RORA deficiency” by SPSS 22 software, and a *P* value of < 0.05 was considered significant.

## Supplementary Information

**Additional file 1: Data S1.** Materials andmethods. **Table S1.** Sequencesof primers for the real time quantitative PCR (qPCR). **Figure S1.**Representative pictures of full blots for Western Blotting. **Figure S2.** Potential effect of SOD mimetic and RORA agonist on maternaldiabetes-mediated gene expression. **Figure S3.** Potentialeffect of SOD mimetic and RORA agonist on maternal diabetes-mediated DNA methylation on the RORA promoter. **Figure S4.** Potential effect of SOD mimetic and RORA agonist onmaternal diabetes-mediated histonemodifications on the RORA promoter. **FigureS5.** Intestine-specific RORA deficiency does not affect maternaldiabetes-mediated autism-like behaviors.

## Data Availability

All data generated or analyzed during this study are included in this published article and its Additioanl files.

## Abbreviations

ALB: Autism-like behavior
ASD: Autism spectrum disorders
ChIP: Chromatin immunoprecipitation
CYP19A1: Cytochrome P450, family 19 (aromatase)
IEC: Intestine epithelial cells
IL1β: Interleukin-1β
MCP1: Monocyte chemoattractant protein-1
PBMC: Peripheral blood mononuclear cells
qPCR: Quantitative real-time PCR
RORA: Retinoic acid-related orphan receptor alpha
ROS: Reactive oxygen species
SOD2: Superoxide dismutase 2
STZ: Streptozotocin

## References

[1] Courchesne E, Gazestani VH, Lewis NE (2020). Prenatal origins of ASD: the when, what, and how of ASD development. Trends Neurosci.

[2] Baron-Cohen S (2020). Foetal oestrogens and autism. Mol Psychiatry.

[3] Baron-Cohen S (2011). Why are autism spectrum conditions more prevalent in males?. PLoS Biol.

[4] Modabbernia A, Velthorst E, Reichenberg A (2017). Environmental risk factors for autism: an evidence-based review of systematic reviews and meta-analyses. Mol Autism.

[5] Rossignol DA, Frye RE (2012). A review of research trends in physiological abnormalities in autism spectrum disorders: immune dysregulation, inflammation, oxidative stress, mitochondrial dysfunction and environmental toxicant exposures. Mol Psychiatry.

[6] Liang Y (2020). Vitamin D deficiency worsens maternal diabetes induced neurodevelopmental disorder by potentiating hyperglycemia-mediated epigenetic changes. Ann N Y Acad Sci.

[7] Liu J (2021). Maternal diabetes-induced suppression of oxytocin receptor contributes to social deficits in offspring. Front Neurosci.

[8] Wang X (2019). Maternal diabetes induces autism-like behavior by hyperglycemia-mediated persistent oxidative stress and suppression of superoxide dismutase 2. Proc Natl Acad Sci USA.

[9] Li L (2018). Prenatal progestin exposure is associated with autism spectrum disorders. Front Psychiatry.

[10] Xie W (2018). Resveratrol ameliorates prenatal progestin exposure-induced autism-like behavior through ERβ activation. Mol Autism.

[11] Zou Y (2017). Prenatal levonorgestrel exposure induces autism-like behavior in offspring through ERβ suppression in the amygdala. Mol Autism.

[12] Xiang D (2020). Berberine ameliorates prenatal dihydrotestosterone exposure-induced autism-like behavior by suppression of androgen receptor. Front Cell Neurosci.

[13] Bjorklund G (2020). Oxidative stress in autism spectrum disorder. Mol Neurobiol.

[14] Bralten J (2018). Autism spectrum disorders and autistic traits share genetics and biology. Mol Psychiatry.

[15] Wang M (2019). Alteration of gut microbiota-associated epitopes in children with autism spectrum disorders. Brain Behav Immun.

[16] Yap CX (2021). Autism-related dietary preferences mediate autism-gut microbiome associations. Cell.

[17] Li Y (2020). The gut microbiota regulates autism-like behavior by mediating vitamin B6 homeostasis in EphB6-deficient mice. Microbiome.

[18] Xu M (2019). Association between gut microbiota and autism spectrum disorder: a systematic review and meta-analysis. Front Psychiatry.

[19] Srikantha P, Mohajeri MH (2019). The possible role of the microbiota-gut-brain-axis in autism spectrum disorder. Int J Mol Sci.

[20] McElhanon BO (2014). Gastrointestinal symptoms in autism spectrum disorder: a meta-analysis. Pediatrics.

[21] Solt LA, Burris TP (2012). Action of RORs and their ligands in (patho)physiology. Trends Endocrinol Metab.

[22] Han YH (2019). A maresin 1/RORalpha/12-lipoxygenase autoregulatory circuit prevents inflammation and progression of nonalcoholic steatohepatitis. J Clin Invest.

[23] Sayad A (2017). Retinoic acid-related orphan receptor alpha (RORA) variants are associated with autism spectrum disorder. Metab Brain Dis.

[24] Hu VW (2015). Investigation of sex differences in the expression of RORA and its transcriptional targets in the brain as a potential contributor to the sex bias in autism. Mol Autism.

[25] Nguyen A (2010). Global methylation profiling of lymphoblastoid cell lines reveals epigenetic contributions to autism spectrum disorders and a novel autism candidate gene, RORA, whose protein product is reduced in autistic brain. FASEB J.

[26] Salehi M (2017). RORA and autism in the Isfahan population: is there an epigenetic relationship. Cell J.

[27] Sarachana T, Hu VW (2013). Genome-wide identification of transcriptional targets of RORA reveals direct regulation of multiple genes associated with autism spectrum disorder. Mol Autism.

[28] Sarachana T (2011). Sex hormones in autism: androgens and estrogens differentially and reciprocally regulate RORA, a novel candidate gene for autism. PLoS ONE.

[29] Han YH (2014). RORalpha decreases oxidative stress through the induction of SOD2 and GPx1 expression and thereby protects against nonalcoholic steatohepatitis in mice. Antioxid Redox Signal.

[30] Liu J (2017). Effect of vitamin A supplementation on gut microbiota in children with autism spectrum disorders—a pilot study. BMC Microbiol.

[31] Lo BC (2016). The orphan nuclear receptor RORalpha and group 3 innate lymphoid cells drive fibrosis in a mouse model of Crohn’s disease. Sci Immunol.

[32] Liang Y (2021). Vitamin D deficiency worsens maternal diabetes induced neurodevelopmental disorder by potentiating hyperglycemia-mediated epigenetic changes. Ann NY Acad Sci.

[33] Kuang J (2014). Identification of insulin as a novel retinoic acid receptor-related orphan receptor alpha target gene. FEBS Lett.

[34] El-Osta A (2008). Transient high glucose causes persistent epigenetic changes and altered gene expression during subsequent normoglycemia. J Exp Med.

[35] Liu Z (2014). Estradiol improves cardiovascular function through up-regulation of SOD2 on vascular wall. Redox Biol.

[36] Zhan Y (2016). ERβ expression in the endothelium ameliorates ischemia/reperfusion-mediated oxidative burst and vascular injury. Free Radic Biol Med.

[37] Jaggar M (2020). You’ve got male: sex and the microbiota-gut-brain axis across the lifespan. Front Neuroendocrinol.

[38] Inoue R (2016). A preliminary investigation on the relationship between gut microbiota and gene expressions in peripheral mononuclear cells of infants with autism spectrum disorders. Biosci Biotechnol Biochem.

[39] Rudolph MD (2018). Maternal IL-6 during pregnancy can be estimated from newborn brain connectivity and predicts future working memory in offspring. Nat Neurosci.

[40] Kim S (2017). Maternal gut bacteria promote neurodevelopmental abnormalities in mouse offspring. Nature.

[41] Nejati Moharrami N (2018). RORalpha controls inflammatory state of human macrophages. PLoS ONE.

[42] Hams E (2020). Role for retinoic acid-related orphan receptor alpha (RORalpha) expressing macrophages in diet-induced obesity. Front Immunol.

[43] Ma S (2020). Alterations in gut microbiota of gestational diabetes patients during the first trimester of pregnancy. Front Cell Infect Microbiol.

[44] De Angelis M (2013). Fecal microbiota and metabolome of children with autism and pervasive developmental disorder not otherwise specified. PLoS ONE.

[45] Hasain Z (2020). Gut microbiota and gestational diabetes mellitus: a review of host-gut microbiota interactions and their therapeutic potential. Front Cell Infect Microbiol.

[46] Eshraghi RS (2020). Gut-induced inflammation during development may compromise the blood–brain barrier and predispose to autism spectrum disorder. J Clin Med.

[47] Chinna Meyyappan A (2020). Effect of fecal microbiota transplant on symptoms of psychiatric disorders: a systematic review. BMC Psychiatry.

[48] Mayer EA, Tillisch K, Gupta A (2015). Gut/brain axis and the microbiota. J Clin Invest.

[49] Fung TC, Olson CA, Hsiao EY (2017). Interactions between the microbiota, immune and nervous systems in health and disease. Nat Neurosci.

[50] Sgritta M (2019). Mechanisms underlying microbial-mediated changes in social behavior in mouse models of autism spectrum disorder. Neuron.

[51] Lee CYQ, Franks AE, Hill-Yardin EL (2020). Autism-associated synaptic mutations impact the gut-brain axis in mice. Brain Behav Immun.

[52] Silverman JL (2010). Behavioural phenotyping assays for mouse models of autism. Nat Rev Neurosci.

[53] Schaafsma SM (2017). Sex-specific gene-environment interactions underlying ASD-like behaviors. Proc Natl Acad Sci USA.

[54] Moy SS (2004). Sociability and preference for social novelty in five inbred strains: an approach to assess autistic-like behavior in mice. Genes Brain Behav.

[55] Lee J (2021). Distinct age-specific miregulome profiling of isolated small and large intestinal epithelial cells in mice. Int J Mol Sci.

[56] Lee J (2015). Profiles of microRNA networks in intestinal epithelial cells in a mouse model of colitis. Sci Rep.

[57] Hsiao EY (2013). Microbiota modulate behavioral and physiological abnormalities associated with neurodevelopmental disorders. Cell.

[58] Tabouy L (2018). Dysbiosis of microbiome and probiotic treatment in a genetic model of autism spectrum disorders. Brain Behav Immun.

[59] Cristiano C (2018). Palmitoylethanolamide counteracts autistic-like behaviours in BTBR T+tf/J mice: contribution of central and peripheral mechanisms. Brain Behav Immun.

[60] Sharon G (2019). Human gut microbiota from autism spectrum disorder promote behavioral symptoms in mice. Cell.

