# Supplementary material for: Maternal diabetes-mediated RORA suppression contributes to gastrointestinal symptoms in autism-like mouse offspring
Source: BMC Neurosci. 2022 Feb 14;23:8. doi: 10.1186/s12868-022-00693-0 (PMC8842926; doi:10.1186/s12868-022-00693-0)
Supplement: Supplementary file 1 — Additional file 1: Data S1. Materials andmethods. Table S1. Sequencesof primers for the real time quantitative PCR (qPCR). Figure S1.Representative pictures of full blots for Western Blotting. Figure S2. Potential effect of SOD mimetic and RORA agonist on maternaldiabetes-mediated gene expression. Figure S3. Potentialeffect of SOD mimetic and RORA agonist on maternal diabetes-mediated DNA methylation on the RORA promoter. Figure S4. Potential effect of SOD mimetic and RORA agonist onmaternal diabetes-mediated histonemodifications on the RORA promoter. FigureS5. Intestine-specific RORA deficiency does not affect maternaldiabetes-mediated autism-like behaviors. [file 12868_2022_693_MOESM1_ESM.docx]

**Maternal Diabetes-Mediated RORA Suppression Contributes to Gastrointestinal Symptoms in Autism-Like Mouse Offspring**

Li Xiao^1,^*, Min Wang^2,^*, Wanhua Zhang^1^, Yuan Song^2^, Jiaying Zeng^1^, Huilin Li^2^,

Hong Yu^1^, Ling Li^2,#^, Pingming Gao^1,#^, Paul Yao^1,2,#^

**Additional file 1**

Data S1. MATERIALS AND METHODS

A detailed description can be found in Data S1, and the related primers used in this study are shown in Table S1.

**Reagents and materials**. The antibodies for β-actin (sc-47778), CYP19 (sc-374176), RORA (sc-518081) and SOD2 (sc-30080) were obtained from Santa Cruz Biotechnology. Antibody for 8-oxo-dG (4354-MC-050) was purchased from Novus Biologicals; Antibodies for acetyl-histone H4 K5, K8, K12, and K16 (H4K5,8,12,16ac, #PA5-40084) were obtained from Invitrogen. Antibodies for histone H3 acetyl K9, K14, K18, K23, K27(H3K9,14,18,23,27ac, ab47915), H4K20me1 (ab9051), H4K20me3 (ab9053), H4R3me1 (ab17339), H3K9me2 (ab1220), H3K9me3 (ab8898), H3K27me2 (ab24684), and H3K27me3 (ab6002) were obtained from Abcam, and 3-nitrotyrosine (3-NT) was measured using the 3-Nitrotyrosine ELISA Kit (ab116691 from Abcam) per manufacturers’ instructions. The mitochondrial fraction was isolated using a Pierce Mitochondria Isolation Kit (Pierce Biotechnology) according to manufacturers’ instructions. Protein concentration was measured using the Coomassie Protein Assay Kit (Pierce Biotechnology). Fluorescein isothiocyanate-labeled dextran (FITC-dextran, #46944), RORA agonist SR1078 (#557352), streptozocin (STZ, #18883-66-4), and MnTBAP, a cell-permeable superoxide dismutase (SOD) mimetic and peroxynitrite scavenger (#475870), were obtained from Sigma.

**RT reaction and real-time quantitative PCR**. Total RNA from treated cells was extracted using the RNeasy Micro Kit (Qiagen), and the RNA was reverse transcribed using an Omniscript RT kit (Qiagen). All the primers were designed using Primer 3 Plus software with the Tm at 60°C, primer size of 21 bp, and product length in the range of 140-160 bp (see Table S1). The primers were validated with an amplification efficiency in the range of 1.9-2.1 and the amplified products were confirmed with agarose gel. Real-time quantitative PCR was run on iCycler iQ (Bio-Rad) with the Quantitect SYBR green PCR kit (Qiagen). The PCR was performed by denaturing at 95°C for 8 min followed by 45 cycles of denaturation at 95°C, annealing at 60°C, and extension at 72°C for 10 s, respectively. 1 µl of each cDNA was used to measure target genes. β-actin was used as the housekeeping gene for transcript normalization, and the mean values were used to calculate relative transcript levels with the ^ΔΔ^CT method per instructions from Qiagen. In brief, the amplified transcripts were quantified by the comparative threshold cycle method using β-actin as a normalizer. Fold changes in gene mRNA expression were calculated as 2^−ΔΔCT^ with CT = threshold cycle, ΔCT=CT (target gene)-CT(β-actin), and the ΔΔCT =ΔCT (experimental)-ΔCT (reference).

**Western blotting.** Cells were lysed in an ice-cold lysis buffer (0.137M NaCl, 2mM EDTA, 10% glycerol, 1% NP-40, 20mM Tris base, pH 8.0) with protease inhibitor cocktail (Sigma). The proteins were separated in 10% SDS-PAGE and transferred to the PVDF membrane, which was then blotted using primary antibodies (1:1000) and then simultaneously incubated with the differentially labeled species-specific secondary antibodies, anti-RABBIT IRDye™ 800CW (green) and anti-MOUSE (or goat) ALEXA680 (red). Membranes were scanned and quantitated using the ODYSSEY Infrared Imaging System (LI-COR, NE) [1].

**Chromatin immunoprecipitation (ChIP).** Cells were washed and crosslinked using 1% formaldehyde for 20 min and terminated by 0.1 M glycine. Cell lysates were sonicated and centrifuged, and 500 µg of protein were pre-cleared by BSA/salmon sperm DNA with preimmune IgG and a slurry of Protein A Agarose beads. Immunoprecipitations were performed with the indicated antibodies, BSA/salmon sperm DNA and a 50% slurry of Protein A agarose beads. Input and immunoprecipitates were washed, eluted, and then incubated with 0.2mg/ml Proteinase K for 2 h at 42˚C, followed by 6 h at 65°C to reverse the formaldehyde crosslinking. DNA fragments were recovered through phenol/chloroform extraction and ethanol precipitation. A ~150 bp fragment on the related promoters was amplified by real-time PCR (qPCR) using the primers provided in Table S1.

**Measurement of ROS generation.** Treated cells were seeded in a 24-well plate and incubated with 10 μM CM-H2DCFDA (Invitrogen) for 45 min at 37°C, and then the intracellular formation of reactive oxygen species (ROS) was measured at excitation/emission wavelengths of 485/530nm using a FLx800 microplate fluorescence reader (Bio-Tek). The data was normalized as arbitrary units [2, 3].

**Measurement of DNA breaks**. Comet assay was conducted using a CometAssay™ kit (Cat No. TA800) from R&D Systems Inc, and the 8-OHdG formation was measured using an OxiSelect™ Oxidative DNA Damage ELISA Kit (Cat No. STA320, from Cell Biolabs Inc.) according to manufacturers’ instructions.

**DNA methylation analysis.** We developed a real-time PCR-based method for methylation-specific PCR (MSP) analysis on the mouse RORA promoter according to the previously described method with some modifications [4, 5]. Genomic DNA from mouse HSC cells was extracted and purified before then being treated by bisulfite modification using the EpiJET Bisulfite Conversion Kit (#K1461, Fisher). The modified DNA was then amplified using methylated and unmethylated primers for MSP that were designed using Methprimer software (<http://www.urogene.org/cgi-bin/methprimer/methprimer.cgi>) with details as follows. Methylated primer: forward 5’- gag tgt ttt aac gcg gat aat c -3’, reverse 5’- aaa aaa aat aaa ata cct cct acg c -3’; unmethylated primer: forward 5’- gag tgt ttt aat gtg gat aat tgg -3’; reverse 5’- aaa aaa ata aaa tac ctc cta cac c -3’. product size: 189bp (methylated) & 188bp (unmethylated); CpG island size: 198bp; Tm: 57.8°C. The final methylation readout was normalized by unmethylated input PCR.

**In vivo mouse experiments.** The animal protocol conformed to US NIH guidelines (Guide for the Care and Use of Laboratory Animals, No. 85-23, revised 1996), and was reviewed and approved by the Institutional Animal Care and Use Committee from Foshan Maternity and Child Health Care Hospital and Hainan Women and Children's Medical Center, and this study is in accordance with the ARRIVE guidelines.

Generation of intestine-specific RORA knockout mice. The RORA^fl/fl^ mouse, which has loxP flanking sites targeting exon 3 of the RORA gene, was generated by in vitro fertilization and was obtained for the study as a generous gift from Dr. Haimou Zhang from Hubei University. The Villin-cre (Vil1-cre) transgenic mice (#021504) have the mouse villin 1 promoter directing expression of Cre recombinase to villus and crypt epithelial cells of the small and large intestines, was obtained from Jackson Laboratories. To generate intestine-specific RORA^-/-^ null mouse (Vil1-cre-RORA^fl/fl^), the RORA^fl/fl^ mice were cross-bred with Vil1-cre mice for over 4 generations on the C57BL/6J background. Positive offspring were confirmed by genotyping through PCR using specific primers (see Table S1) for the presence of both loxP sites within RORA alleles and Cre recombinase [6].

Generation of diabetic mice. All the experimental mice were either RORA wild type (WT) or RORA null (RORA^-/-^) mice with a C57BL/6J mixed genetic background. In the generation of diabetic mice, adult (3-month-old) female mice with either WT or RORA^-/-^ backgrounds were monitored for estrous cycles with daily vaginal smears. Only mice with at least two regular 4 to 5-day estrous cycles were included in the studies. Chronic diabetic female mice were induced by injection of 35 mg/kg streptozocin (STZ, 0.05 M sodium citrate, pH 5.5) after an 8-hour fasting period. Animals with blood glucose >250mg/dl were considered positive with a success rate of ~90%, while control (CTL) mice received only vehicle injection.

Mouse Protocol 1: Prenatal treatment of diabetes or chemicals. Verified pregnant dams (n=9 for each group) were randomly assigned to the following 4 groups: Group 1: CTL group mice receiving only vehicle treatment (CTL/VEH); Group 2: STZ mice receiving only vehicle treatment (STZ/VEH); Group 3: CTL group mice receiving 10mg/kg/day of MnTBAP (dissolved in DMSO) injection (CTL/MnTBAP); Group 4. STZ mice receiving 10mg/kg/day of RORA agonist SR1078 (dissolved in DMSO) injection (STZ/SR1078). The injection was conducted on days 1, 4, 7, 10, 13, 16 and 19 of pregnancy, respectively. Neurons from the amygdala were isolated on embryonic day 18 (E18) as described below. The male offspring (1 male offspring from each dam was randomly selected for experiments) were separated from the dams on day 21, fed with normal chow, and underwent behavior tests until 7-8 weeks of age. GI symptoms were evaluated, the whole blood was collected by heart puncture and the peripheral blood mononuclear cells (PBMC) were isolated, and the intestine epithelial cells (IEC) were isolated as described below for further biomedical analysis,

Mouse Protocol 2: Prenatal treatment of diabetes or RORA deficiency. Verified pregnant dams (n=9 for each group) were randomly assigned to the following 4 groups. Group 1: CTL group mice with RORA WT background (CTL/WT); Group 2: STZ mice with RORA WT background (STZ/WT); Group 3: CTL group mice with intestine-specific RORA knockdown background (CTL/RORA^-/-^); Group 4: STZ mice with intestine-specific RORA knockdown background (STZ/RORA^-/-^). The subsequent offspring were used for analysis as described in Mouse Protocol 1.

**Animal behavior test.** The animal behavior test of offspring was carried out at 7-8 weeks of age. Autism-like behavior was evaluated using ultrasonic vocalization (USV), social interaction (SI) tests and a three-chambered social test as described below [7-9].

Ultrasonic vocalizations (USV). The USV test was conducted on postnatal day 7 during a maternal-separation paradigm. The individual pup was isolated and placed in the sound-proof chambers, and the USV was recorded by an externally polarized condenser microphone (30-300 kHz) that was attached 15-20 cm above the floor of isolation chamber, and the microphone was connected to Avisoft-UltrasoundGate recording software (from Avisoft Bioacoustics) and the pup-emitted calls were transferred to WAV sound files. Recorded number of USV was analyzed by a generalized linear model through a negative binomial distribution and a log-link function.

Social interaction (SI) test. The social interaction test was performed in an open-field arena using the Open Field Starter Package for Mouse (Cat#: MED-OFAS-MSU, Med Associates Inc.). The subjects, including Test and Stranger mice, were habituated in the arena separately for 5 min before the test. During the test, the mice were placed into the apparatus for 20 min and the time spent following, mounting, grooming, and sniffing any body parts of the other mouse was counted as the indicator of social engagement, and the social interaction time was quantitated and analyzed by EthoVision XT animal tracking software [10]. Each “Stranger” mouse during the test was used only once, and matches with the same sex, weight, and age, and had no previous contact with the test mouse [11-14].

Three-chambered social test. 7-8 weeks old mice were employed to determine the sociability and the preference for social novelty. Target subjects, including Stranger 1 and Stranger 2, were placed inside the wire cages for 3 days before the beginning of testing, and the test mice were placed in the testing room for at least 45 min before the start of behavioral tests. For the sociability test, the test mouse was placed to the middle chamber and left to habituate for 5 min, after which an unfamiliar Stranger 1 mouse was introduced into a wire cage in one of the side-chambers and an empty wire cage on the other side-chamber. The test mouse was allowed to explore freely for all 3 chambers for 10min. Following this, a novel Stranger 2 mouse was introduced into the previously empty wire cage and the test mouse was again left to explore freely for 10 min. All the parameters, including time spent in each chamber, number of entries into the chambers and track maps, were recorded and calculated by automated SMART software.

**SOD2 activity assay.** SOD2 was obtained from the mitochondrial fraction that was isolated using a Pierce Mitochondria Isolation Kit (Pierce) according to manufacturers’ instructions. SOD activity was measured as described previously [15]. In brief, a stable O2.- source was generated through the conversion action of XOD (xanthine oxidase) from xanthine and was mixed with chemiluminescent (CL) reagents to achieve a stable light emission. The SOD2 sample injection can scavenge O2.- and the subsequent decrease of chemiluminescent response is proportional to the SOD2 activity. This system can have a detection limit of 0.001U/ml with the linear range of 0.03~2.00 U/ml. The results were normalized by protein concentration and expressed as Units/mg proteins (U/mg) [16].

**In vitro primary culture of amygdala neurons**. Amygdala tissues were dissected from mice on embryonic day 18 (E18 mouse). Tissues were treated with 0.05% trypsin EDTA for 15 min at 37°C. Trypsin EDTA was replaced with soybean trypsin inhibitor (Sigma) for 5 min at 37°C to stop the reaction. This was then replaced with supplemented Neurobasal A (Invitrogen) followed by mechanical dissociation. Cells were then resuspended in culture media, including Neurobasal A, B27, 1×GlutaMAX and 100 U/ml Pen/Strep (from Invitrogen), and then the cells were incubated at 37°C and 5% CO2. The isolated amygdala neurons were then used for immunostaining [13].

**Isolation of mouse PBMC cells**. The heparinized peripheral blood was collected from mouse subjects by heart puncture and was diluted 1:3 with Hank's balanced salts solution without Ca^2+^/Mg^2+^ (HBSS solution). The diluted blood was layered onto 10 ml of Ficoll-Paque in 15 ml sterile centrifuge tubes followed by centrifugation at 300×g at 20ºC for 40 min. The PBMC layers were then harvested and washed by HBSS solution. The pellets were then resuspended with lysing buffer containing 150 mM NH4Cl, 1.0 mM KHCO3, and 0.1 mM Na2EDTA, pH 7.4 and incubated for 5 min at room temperature to remove contaminated red cells. The cell suspensions were then centrifuged and washed with HBSS solution before the cell pellet was resuspended for further biomedical analysis.

**Isolation of mouse intestine epithelial cells (IEC).** The protocol for isolation of IEC cells was based on the previously described method with minor modifications. In brief, the small and large intestines were harvested individually from treated mice and rinsed extensively with RPMI-1640 media (from Lonza) after Peyer’s patches were removed (for small intestine). The rinsed intestines were opened longitudinally and macerated; the tissue pieces were shaken gently in RPMI-1640 containing 2 mM EDTA and 10% fetal calf serum. The tissue preparations were passed through 70-μm mesh filters, and the resulting single-cell suspensions were applied to Percoll (from Sigma) density gradients of 25%, 40%, and 75%. After centrifugation at 2,000×g for 20 min, the interface between the 25% and 40% layers was collected to obtain IECs. The cells were stained using antibodies for either epithelial cell adhesion molecule (EpCAM, from Biolegend) or CD45 (from Biolegend) and nucleic acid dye (Via-Probe, from BD Biosiences). The Via-Probe^-^/CD45^-^/EpCAM^+^ IEC were sorted using BD FACSMelody^TM^ Cell Sorter (BD Biosciences) for further biomedical analysis [17, 18].

**Intestinal permeability assay.** The protocol was followed based on the previously described method with minor modifications. In brief, treated mice were fasted for 4 h before the experiment and then the FITC-dextran (50mg/mL, Cat# 46944 from Sigma) was gavaged to mice (600 mg/kg). After 4 h, the whole blood was collected by cardiac puncture and placed at room temperature for 1 h before being centrifuged at a speed of 3000 rpm for 10 min. The supernatant was then transferred to a new tube for further centrifugation at a speed of 12,000 rpm for 10 min at 4 °C. The subsequent supernatant (serum) was diluted with equal volume of PBS and 100 μL diluted serum was added to a 96-cell microplate. The concentration of FITC in serum was determined at excitation/emission wavelengths of 485/530nm using a FLx800 microplate fluorescence reader (Bio-Tek). The serial diluted FITC-dextran (0, 0.5, 1, 2, 4, 6, 8, 10 μg/μL) was used as standards. Serum of mice administered with PBS was used as negative controls [19, 20].

**Fecal microbiome analysis.** Fecal samples of the experimental mice were collected and stored at −80°C before being processed. The microbial DNA was extracted using a QIAamp Fast DNA Stool Mini Kit (from Qiagen) according to the manufacturer's protocol [21]. The purity and concentration of the extracted DNA were detected using agarose gel electrophoresis. Fecal microbiota was studied by performing V3-V4 16S rDNA amplicon sequencing in order to obtain the operational taxonomic units (OTU) defining the bacterial communities [22]. Sequencing samples from frozen fecal pellets were prepared, sequenced and subsequently processed using the MiSeq Pe300 Sequencing Platform (from Illumina) by Shanghai OE Biotech Inc. The raw data were treated and processed using a QIIME 2^TM^ software package, and the subsequent sequences of OTU were blasted in the Silva database (version 138). The alpha diversity and beta diversity were analyzed using QIIME 2^TM^ software package [19].

**Analysis of cytokines.** Mouse cytokines from either the serum or cell supernatant, including IL1β, IL6, IL17a and MCP1 were measured using the Bio-Plex Pro Mouse Cytokine 23-plex Assay kit (#M60009RDPD from BioRad) and Bio-Plex 200 Systems (BioRad) according to the manufacturer’s instructions. Protein concentration in lysates was determined by Coomassie Protein Assay Kit (Pierce Biotechnology) according to the manufacturer’s instructions and lysates were adjusted to 200 mg/ml with extraction buffer. 50 mL lysate were diluted 2× in sample dilution buffer and analyzed in duplicates. Analytes were quantified in each sample against a calibration curve of known concentrations [23].

**Immunostaining**. The treated cells were transferred to cover slips and the cells were fixed in 4% paraformaldehyde for 20 min before being incubated with 0.3% Triton X-100 in PBS for 15 min. After blocking with 5% goat serum in PBS at room temperature for 30 min, cells were incubated with antibody for either RORA or 8-oxo-dG (# 4354-MC-050, from Novus Biologicals) for 12 h at 4°C and subsequently with secondary antibody Alexa Fluor 488. The cover slips were then mounted by antifade Mountant with DAPI (staining nuclei, in blue). The photographs were taken using a Confocal Laser Microscope (Leica, 20x lens) and quantitated by Image J. software [24].

**Statistical analysis**. The data was given as mean ± SD, and all the experiments were performed at least in quadruplicate unless indicated otherwise. The one-way analysis of variance (ANOVA) followed by the Tukey−Kramer test was used to determine statistical significance of different groups, and the two-way ANOVA followed by the Bonferroni post hoc test was used to determine the differences of two factors (e.g. RORA deficiency and maternal diabetes) by SPSS 22 software, and a *P* value of <0.05 was considered significant.

REFERENCES

1. Ceradini, D.J., et al., *Decreasing intracellular superoxide corrects defective ischemia-induced new vessel formation in diabetic mice.* J Biol Chem, 2008. **283**(16): p. 10930-8.

2. Zhang, H., et al., *Combination of betulinic acid and chidamide inhibits acute myeloid leukemia by suppression of the HIF1alpha pathway and generation of reactive oxygen species.* Oncotarget, 2017. **8**(55): p. 94743-94758.

3. Yao, D., et al., *Fatty acid-mediated intracellular iron translocation: a synergistic mechanism of oxidative injury.* Free Radic Biol Med, 2005. **39**(10): p. 1385-98.

4. Eads, C.A., et al., *MethyLight: a high-throughput assay to measure DNA methylation.* Nucleic Acids Res, 2000. **28**(8): p. E32.

5. Nosho, K., et al., *Comprehensive biostatistical analysis of CpG island methylator phenotype in colorectal cancer using a large population-based sample.* PLoS ONE, 2008. **3**(11): p. e3698.

6. Han, Y.H., et al., *A maresin 1/RORalpha/12-lipoxygenase autoregulatory circuit prevents inflammation and progression of nonalcoholic steatohepatitis.* J Clin Invest, 2019. **129**(4): p. 1684-1698.

7. Silverman, J.L., et al., *Behavioural phenotyping assays for mouse models of autism.* Nat Rev Neurosci, 2010. **11**(7): p. 490-502.

8. Schaafsma, S.M., et al., *Sex-specific gene-environment interactions underlying ASD-like behaviors.* Proc Natl Acad Sci U S A, 2017. **114**(6): p. 1383-1388.

9. Moy, S.S., et al., *Sociability and preference for social novelty in five inbred strains: an approach to assess autistic-like behavior in mice.* Genes Brain Behav, 2004. **3**(5): p. 287-302.

10. Mufford, J.T., et al., *The development of a non-invasive behavioral model of thermal heat stress in laboratory mice (Mus musculus).* J Neurosci Methods, 2016. **268**: p. 189-95.

11. Bahi, A., *Sustained lentiviral-mediated overexpression of microRNA124a in the dentate gyrus exacerbates anxiety- and autism-like behaviors associated with neonatal isolation in rats.* Behav Brain Res, 2016. **311**: p. 298-308.

12. Bahi, A., *Hippocampal BDNF overexpression or microR124a silencing reduces anxiety- and autism-like behaviors in rats.* Behav Brain Res, 2017. **326**: p. 281-290.

13. Zou, Y., et al., *Prenatal levonorgestrel exposure induces autism-like behavior in offspring through ERβ suppression in the amygdala.* Mol Autism, 2017. **8**: p. 46.

14. Xie, W., et al., *Resveratrol ameliorates prenatal progestin exposure-induced autism-like behavior through ERβ activation.* Mol Autism, 2018. **9**: p. 43.

15. Yao, D., et al., *Chemiluminescence detection of superoxide anion release and superoxide dismutase activity: modulation effect of Pulsatilla chinensis.* Anal Bioanal Chem, 2004. **379**(1): p. 171-7.

16. Kong, D., et al., *SIRT1-mediated ERbeta suppression in the endothelium contributes to vascular aging.* Aging Cell, 2016.

17. Lee, J., et al., *Distinct Age-Specific miRegulome Profiling of Isolated Small and Large Intestinal Epithelial Cells in Mice.* Int J Mol Sci, 2021. **22**(7).

18. Lee, J., et al., *Profiles of microRNA networks in intestinal epithelial cells in a mouse model of colitis.* Sci Rep, 2015. **5**: p. 18174.

19. Li, Y., et al., *The gut microbiota regulates autism-like behavior by mediating vitamin B6 homeostasis in EphB6-deficient mice.* Microbiome, 2020. **8**(1): p. 120.

20. Hsiao, E.Y., et al., *Microbiota modulate behavioral and physiological abnormalities associated with neurodevelopmental disorders.* Cell, 2013. **155**(7): p. 1451-63.

21. Tabouy, L., et al., *Dysbiosis of microbiome and probiotic treatment in a genetic model of autism spectrum disorders.* Brain Behav Immun, 2018. **73**: p. 310-319.

22. Cristiano, C., et al., *Palmitoylethanolamide counteracts autistic-like behaviours in BTBR T+tf/J mice: Contribution of central and peripheral mechanisms.* Brain Behav Immun, 2018. **74**: p. 166-175.

23. Sharon, G., et al., *Human Gut Microbiota from Autism Spectrum Disorder Promote Behavioral Symptoms in Mice.* Cell, 2019. **177**(6): p. 1600-1618 e17.

24. Wang, X., et al., *Maternal diabetes induces autism-like behavior by hyperglycemia-mediated persistent oxidative stress and suppression of superoxide dismutase 2.* Proc Natl Acad Sci U S A, 2019. **116**(47): p. 23743-23752.

**Table S1. Sequences of primers for the real time quantitative PCR (qPCR)**

| Gene | Species | Analysis | Forward primer (5'→3') | Reverse primer (5'→3') |
| --- | --- | --- | --- | --- |
| RORA | Mouse | ChIP | taaccggatttgtctcctcct | cgatcctccctctcctcttta |
| SOD2 | Mouse | ChIP | aggtcactccgggcataaat | agctgcaaagcttccactcta |
| β-actin | Mouse | mRNA | tcttgggtatggaatcctgtg | atctccttctgcatcctgtca |
| RORA | Mouse | mRNA | attggacatcaatgggatcaa | tttggatatgttctgggcaag |
| CYP19A1 | Mouse | mRNA | tatgaacgatccgtcaaggac | ttctctttcgtcaggtctcca |
| SOD2 | Mouse | mRNA | ggcctacgtgaacaatctcaa | tcaggtttgtccagaaaatgg |
| loxP | Mouse | Genotype | ttgtgtataccaccacaagtgcacc | agtacaggacacttcggtgtctacc |
| Cre | Mouse | Genotype | cttgggctgccagaatttctc | cccagaaatgccagattacg |

FIGURE S1

**Figure S1. Representative pictures of full blots for Western Blotting.** (a) Full blots for Figure 1c. (b) Full blots for Figure 4c. (c). Full blots for Figure 6c.

FIGURE S2

**Figure S2. Potential effect of SOD mimetic and RORA agonist on maternal diabetes-mediated gene expression**. Dams were treated by control (CTL/VEH), diabetes (STZ/VEH), diabetes plus SOD mimetic MnTBAP (STZ/MnTBAP), or diabetes plus RORA agonist SR1078 (STZ/SR1078) during pregnancy, and the subsequent 7-8 weeks old male offspring were sacrificed, and the tissues for hypothalamus and hippocampus were isolated for mRNA analysis. (a) mRNA levels in hypothalamus, n=4. (b) mRNA levels in hippocampus, n=4. The one-way ANOVA analysis was performed to determine statistical significance of different groups. *, *P*<0.0001, vs CTL/VEH group. Data were expressed as mean ± SD.

FIGURE S3

**Figure S3. Potential effect of SOD mimetic and RORA agonist on maternal diabetes-mediated DNA methylation on the RORA promoter.** Dams were treated by control (CTL/VEH), diabetes (STZ/VEH), diabetes plus SOD mimetic MnTBAP (STZ/MnTBAP), or diabetes plus RORA agonist SR1078 (STZ/SR1078) during pregnancy, and the amygdala neurons were isolated for DNA methylation on the RORA promoter, n=4. The one-way ANOVA analysis was performed to determine statistical significance of different groups. Data were expressed as mean ± SD.

FIGURE S4

**Figure S4. Potential effect of SOD mimetic and RORA agonist on maternal diabetes-mediated histone modifications on the RORA promoter.** Dams were treated by control (CTL/VEH), diabetes (STZ/VEH), diabetes plus SOD mimetic MnTBAP (STZ/MnTBAP), or diabetes plus RORA agonist SR1078 (STZ/SR1078) during pregnancy, and the amygdala neurons were isolated for ChIP analysis. (a) Histone H4 methylation on the RORA promoter, n=4. (b) Histone acetylation on the RORA promoter using H3K9,14,18,23,27ac and H4K5,8,12,16ac antibodies, n=4. The one-way ANOVA analysis was performed to determine statistical significance of different groups. Data were expressed as mean ± SD.

FIGURE S5

**Figure S5.** **Intestine-specific RORA deficiency does not affect maternal diabetes-mediated autism-like behaviors**. Dams from either the control (CTL) or diabetes (STZ) groups were crossbred with either wild type (WT) or intestinal epithelial-specific RORA knockout (RORA^-/-^), and the subsequent male offspring were used for evaluation of autism-like behaviors. (a) Ultrasonic vocalization, n=9. (b) Social interaction (SI) test, with total interaction time and amount of time spent following, mounting, grooming, and sniffing any body parts of the other mouse being calculated, n=9. (c,d) Three-chambered social tests, n=9. (c) Time spent in chamber for sociability. (d) Time spent in chamber for social novelty. The two-way ANOVA analysis was performed to determine statistical significance of different groups. ***, *P*<0.0001; **, *P*<0.001; *, *P*<0.01; N/S, no significance. Data were expressed as mean ± SD.
